# Predictive performance of MRI and CT radiomics in predicting the response to induction chemotherapy in nasopharyngeal carcinoma: a network meta-analysis

**DOI:** 10.3389/fonc.2025.1590420

**Published:** 2025-11-13

**Authors:** Yongjie Jian, Jiaxuan Peng, Gan Yang, Xiaojuan He, Jing Wang, Jun Yin, Hui Shi, Di Tao, Qiyu Lan, Zuogang Yang, Zhenyu Shu

**Affiliations:** 1 Department of Radiology, Affiliated Hospital of Sichuan Nursing Vocational College, The Third People’s Hospital of Sichuan Province, Chengdu, Sichuan, China; 2 Jinzhou Medical University Postgraduate Training Base (Zhejiang Provincial People’s Hospital, People’s Hospital of Hangzhou Medical College), Hangzhou, Zhejiang, China; 3 Center for Rehabilitation Medicine, Department of Radiology, Zhejiang Provincial People’s Hospital, Affiliated People’s Hospital, Hangzhou Medical College, Hangzhou, Zhejiang, China; 4 Department of General Surgery, Shehong People’s Hospital, Suining, Sichuan, China; 5 Department of Medical Technology, Sichuan Nursing Vocational College, Chengdu, Sichuan, China; 6 Department of Oncology, Affiliated Hospital of Sichuan Nursing Vocational College, The Third People’s Hospital of Sichuan Province, Chengdu, Sichuan, China; 7 Department of Ultrasound, Affiliated Hospital of Sichuan Nursing Vocational College, The Third People’s Hospital of Sichuan Province, Chengdu, Sichuan, China; 8 Department of Radiology, Rangtang Country People’s Hospital, Aba Tibetan and Qiang Autonomous Prefecture, Sichuan, China

**Keywords:** nasopharyngeal carcinoma, induction chemotherapy, radiomics, deep learning, network meta-analysis, machine learning, Bayesian

## Abstract

**Objectives:**

To evaluate the accuracy of different radiomics methods in predicting the response of nasopharyngeal carcinoma (NPC) to induction chemotherapy (IC).

**Methods:**

A systematic search was conducted in PubMed, Embase, Web of Science, and Cochrane Library. Radiomics studies utilizing CT and MRI were included in this network meta-analysis. The quality of the studies was appraised via the PROBAST, RQS, and IBSI guidelines. The sensitivity, specificity, and accuracy of different radiomics models were analyzed.

**Results:**

Ten eligible studies involving 1550 subjects were included. The pooled sensitivity and specificity of the radiomics models were 0.86 (95% CI: 0.78-0.91) and 0.69 (95% CI: 0.62-0.75), respectively. The AUC based on the SROC curve was 0.83 (95% CI: 0.70-0.91). The predictive performance of each model was rated using SUCRA values. The MRI-based support vector machine radiomics model had the highest specificity, and accuracy, at 80.7% and 73.2%, respectively. The MRI-based SVM radiomics combined with clinical features model had the highest sensitivity (82.0%). Among the CT methods, the deep learning (DL)-based convolutional neural network model had the highest sensitivity, and accuracy, at 51.0% and 44.9%, respectively. The PROBAST showed that 7 studies were at risk for bias.

**Conclusion:**

This study synthesized existing evidence to confirm that radiomics serves as a viable exploratory tool for predicting IC efficacy in NPC. MRI-based nonlinear models and clinical-radiomics fusion models exhibit considerable promise, whereas clinical translation necessitates three critical steps: (1) standardized protocols following IBSI/METRICS/RQS guidelines; (2) prospective multicenter validation; and (3) investigating tumor microenvironment mechanisms. These measures will facilitate the transition of radiomics from technical exploration to clinical utility.

**Systematic Review Registration:**

https://www.crd.york.ac.uk/prospero/, identifier CRD42024509331.

## 1 Introduction

Nasopharyngeal carcinoma (NPC), found mostly in Asia, is a form of head and neck cancer originating from the epithelium of the nasopharynx (1), with approximately 75% of cases being diagnosed at the locally advanced stage (2). Induction chemotherapy (IC) is also called neoadjuvant chemotherapy (3). According to the 2022 National Comprehensive Cancer Network (NCCN) guidelines, IC plus concurrent chemoradiotherapy (CCRT) has been listed as a level 1 recommendation for locoregionally advanced NPC (LA-NPC) patients (4). Several retrospective studies have investigated the effectiveness of IC plus intensity-modulated radiotherapy (IMRT) vs. IC plus CCRT for LA-NPC, but the conclusions have been mixed (5–8). Despite the use of standard radiotherapy and chemotherapy following the guidelines on tumor-node-metastasis (TNM) stage, the 5-year treatment failure rate of LA-NPC is still up to 30% (9). This may be because the TNM staging system is only based on anatomical information provided by imaging and ignores intratumoral heterogeneity, resulting in an inability to accurately stratify the risk of LA-NPC (10, 11). Therefore, in clinical practice, identifying the heterogeneity of characteristics within tumors and screening patients who are sensitive to IC are essential.

Radiomics noninvasively characterizes tumor heterogeneity by transforming medical images into high-dimensional quantitative features (12–15). Relevant studies have shown that radiomics can be used to noninvasively evaluate the IC response and provide additional benefits for NPC patients (16–21). Machine learning (ML) serves as the foundational analytical framework, among which the support vector machine (SVM) is a typical method of ML (22). Fully connected neural network (FCNN) model complex nonlinear relationships through simulated biological propagation (23, 24). Deep learning (DL) (a subset of ML) (25), where convolutional neural networks (CNN) excel in medical image pattern recognition. These approaches (SVM/FCNN/CNN) have been successfully implemented for NPC staging, treatment response prediction, and outcome prognostication (26–31), underscoring the translational potential of ML-driven radiomics in precision NPC oncology.

Systematic reviews/meta-analyses of radiomics-based prognostic studies in NPC have validated the utility of radiomics, yet their limitations warrant emphasis. Deng et al. (32) reported that the combined AUC value of radiomics for predicting NPC prognosis was 0.8265. Wang et al. (33) evaluated MRI-based radiomics for predicting local recurrence-free survival (LRFS), distant metastasis-free survival (DMFS), progression-free survival (PFS), and overall survival (OS). Meanwhile, Lee et al. (34) focused on the performance of MRI-based radiomics in PFS prediction, yielding a pooled c-index of 0.762. Yang et al. (31) focus on evaluating radiomics for assessing induction chemotherapy (IC) efficacy in NPC, the overall AUC was 0.91. Collectively, existing research is limited by high heterogeneity, potential biases, data gaps in key subgroups, and retrospective designs, which may undermine the generalizability of conclusions.

This study rigorously assessed the methodological quality of included studies and conducted the first network meta-analysis (NMA) to compare the predictive efficacy of diverse radiomics algorithms for IC efficacy in NPC, thereby establishing a hierarchy of evidence to support clinical decision-making for response-adaptive therapy.

## 2 Materials and methods

### 2.1 Program and registration

This study was conducted according to the Preferred Reporting Items for Systematic Reviews and Meta-Analyses (PRISMA) guidelines (35). The original study protocol was registered with PROSPERO before initiating the systematic search as *a priori* study design (CRD42024509331). The study utilized existing published data, eliminating the need for additional ethical approval and informed consent procedures.

### 2.2 Literature search

The PubMed, Embase, Web of Science, and Cochrane Library databases were searched from inception until May 25, 2024. The search was conducted independently by two researchers (H.S. and Q.L.) following the Cochrane Handbook for Systematic Reviews of Interventions, and discrepancies or uncertainties in the articles were resolved through discussion or consultation with a third reviewer (J.P.). Literature was searched manually via different combinations of free words and MeSH terms. The search terms included “nasopharyngeal carcinoma”, “machine learning”, “deep learning”, “radiomics”, and their variations. See **Supplementary Material Table S1** for a detailed search strategy for MeSH terms.

### 2.3 Inclusion and exclusion criteria

Inclusion criteria: Patients who were diagnosed with NPC and who received IC, regardless of age, sex, race, or country. Research type: Imaging analysis using radiomics to predict the response to IC in NPC. Results: Sensitivity, specificity, and accuracy. Exclusion criteria: Insufficient data integrity to extract two-by-two data tables, conference reports, systematic reviews, summary articles, non-English articles, or conference proceedings.

### 2.4 Data extraction

The following data were extracted from the included articles in a standardized format: (1) study characteristics (first author, year of publication, nationality, affiliation, study period, and study design); (2) cohort characteristics (mean age, patient numbers, including the numbers of patients in the training and validation cohorts, sex, cancer stages, number of patients with effective treatment, and examination methods); and (3) image feature analysis (segmentation software, segmentation method, radiomics software, feature selection method, type of features, number of image features selected, type of validation, and type of algorithm).

For each study, true positive (TP), false positive (FP), false negative (FN), and true negative (TN) values were extracted. TP was defined as no response to treatment, and TN was defined as effective treatment. If there were multiple imaging models of different types in a study, among the models of the same type, the model with the highest AUC value that could extract data was selected. The performance metrics of the external validation cohort (or internal validation cohort, if the former was absent) were recorded, and a two-by-two table was constructed. If the study did not report these values, a two-by-two table from the diagnostic estimates presented in the article for each index test was constructed.

### 2.5 Quality assessment

The Prediction Model Risk of Bias Assessment Tool (PROBAST) checklist was used to assess the risk of bias and applicability (36). Two authors (H.S. and Q.L.) independently assessed the presence of bias and concerns regarding the applicability of the studies, and any differences were resolved by consensus or with the participation of a third reviewer (J.P.) if necessary. The radiomics quality score (RQS) (37) was also applied by two authors (H.S. and Q.L.) to gauge the methodological soundness of the radiomics studies, encompassing image acquisition to validation, ensuring the dependability of the findings. The IBSI guideline provides a comprehensive and unified reporting checklist for radiomics studies (38). Since many items in the IBSI checklist overlap with those in the RQS checklist, two authors (H.S. and Q.L.) only evaluated items relevant to image pre-processing steps.

### 2.6 Statistical analysis

This NMA was conducted within a Bayesian framework via Markov chain Monte Carlo subset simulations (39) according to the PRISMA NMA guidelines (40), and the data of the different models included in the study were directly and indirectly compared. First, traditional meta-analysis was conducted via a bivariate model of pooled sensitivity and specificity, which was visualized via forest plots and summary receiver operating characteristic (SROC) curves. Heterogeneity between the included studies was assessed by Cochrane’s Q test and *I*^2^, with *I*^2^ > 75% indicating greater heterogeneity (41). To further investigate the sources of heterogeneity, meta-regression and subgroup analyses were performed. The following factors were considered as potential sources of heterogeneity: device type (MRI vs. CT), events per variable (EPV) (≥10 vs. <10), ROI structure (2D vs. 3D), model validation (external validation vs. others), algorithms (linear vs. nonlinear), feature quantity after feature dimensionality reduction (≥10 vs. <10), RQS score (≥16.2 vs. <16.2), and sample size (≥200 vs. <200). The network was next drawn, where nodes represent each method and lines represent direct comparisons. The node size was correlated with the number of studies. The line thickness represents the number of comparisons between two studies. The control group was established as the clinical gold standard based on RECIST 1.1 criteria, primarily serving dual purposes: (1) evaluating lesion remission status following IC and (2) Providing a benchmark for comparative validation of predictive models. The data likelihood was specified using a binomial distribution to model the TP and TN counts for each study; a logit link function was employed to transform the accuracy metrics; and the random effects covariance structure was modeled via a multivariate normal distribution to characterize the correlation between sensitivity and specificity. We implemented the node-splitting method to estimate the consistency between the direct and indirect evidence for the whole study and then chose the consistency model or the inconsistency model based on the results (42). To estimate the predictive performance and ranking probability for each model, we used the surface under the cumulative ranking curve (SUCRA). The SUCRA percentages range from 0 to 1, with higher values indicating a greater likelihood of being the best prediction model (43). The predictive performance of the different models was judged by analyzing the sensitivity, specificity, and accuracy indicators. We tested the robustness of the results by sensitivity analysis. In addition, we constructed comparison-adjusted funnel plots and Deek’s test to assess potential publication bias.

Traditional meta-analysis was performed using the “midas” and “metan” package in Stata (version 17.0; Stata Corporation, College Station, Texas, USA). Bayesian NMA and associated graphical analyses were implemented via the “network” package within the same Stata framework.

### 2.7 Evaluation criteria for IC

The response evaluation of IC in all included studies was based on the RECIST version 1.1 (44). Complete response (CR) and partial response (PR) were defined as effective treatment, whereas stable disease (SD) and progressive disease (PD) were defined as no response to treatment. According to this standard, a control group (the gold standard) was established to evaluate whether the lesion was in remission and to compare and verify different models.

## 3 Results

### 3.1 Literature selection

**Figure 1:**
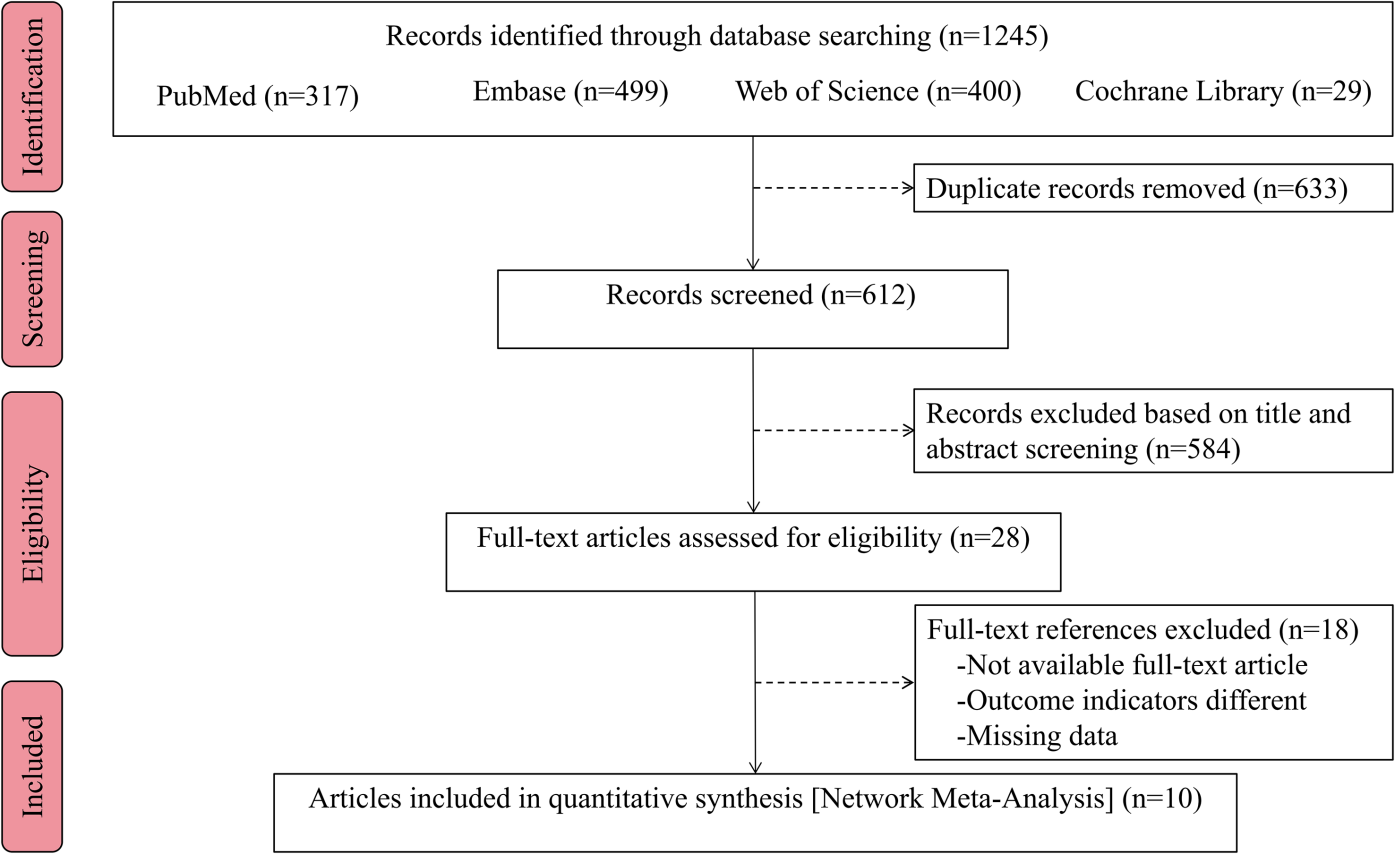
Flow chart of the study selection process.

The initial search for this study yielded a total of 1245 index records. After removing 633 duplicate articles, further review focused on titles and abstracts and excluded an additional 584 unrelated articles. The remaining 28 articles were subsequently evaluated more rigorously, including the accessibility of the full-text version and the feasibility of data extraction, and 10 full-text studies that met the predetermined inclusion criteria were identified for evaluation. The PRISMA flowchart of the selection process is shown in **Figure 1**.

### 3.2 Baseline characteristics of the included studies

These 10 articles included data from 1550 subjects. The baseline characteristics of the included studies are summarized in **Table 1**. Imaging methods included MRI and CT. The models were divided into the following seven types according to different features: (1) the radiomics combined with linear algorithms (logistic regression, LASSO) was used to construct a Radiomics model; (2) the SVM-based radiomics method was used to construct an SVM model; (3) the FCNN-based radiomics method was used to construct a FCNN model; (4) the pretrained CNN of DL was used to construct a CNN model; (5) the radiomics combined with linear algorithms and clinical features was used to construct a Radiomics-Clinical model; (6) the SVM-based radiomics combined with clinical features was used to construct an SVM-Clinical model; and (7) the SVM-based radiomics combined with CNN was used to construct an SVM-CNN model. According to the imaging methods and model types, the following 9 prediction models were included in this study: the MRI Radiomics model, the MRI Radiomics-Clinical model, the MRI SVM model, the MRI SVM-Clinical model, the MRI FCNN model, the CT SVM model, the CT SVM-Clinical model, the CT CNN model, and the CT SVM-CNN model.

**Table 1:** Baseline characteristics of the included studies.

First author	Year	Country	Affiliation	Study period	Study design	Mean Age, (Year)	Number of patients (Training/Validation)	Sex (Male/Female)	tumor stage	Number of patients with effective treatment	Imaging method
Piao (20)	2021	China	Cancer Hospital of the University of Chinese Academy of Sciences	January 2016 to December 2016	R	54(22-70)^*^	108	80/28	III-IV	52	MRI
Wang (17)	2022	China	Sichuan Cancer Hospital & Institute	January 2009 to December 2014 , June 2016 to February 2018	R	47.22 ± 11.44	165 (85/80)	119/46	III-IVb	116	MRI
Wang (19)	2018	China	Guangdong General Hospital	August 2009 to May 2016	R	46.81 ± 10.89	120	95/25	II-IV	70	MRI
Zhang (18)	2020	China	Cancer Hospital of the University of Chinese Academy of Sciences	January 2018 to April 2020	R	42(18-68)^*^	265 TP group (106/44) GP group (81/34)	115/150	II-IVb	125	MRI
Zhao (27)	2020	China	Xijing Hospital	January 2012 to December 2016	R	48.16 ± 10.47	123 (100/23)	36/87	III-IVb	34	MRI
Yang (31)	2022	China	West China Hospital	January 2012 to December 2018	R	52.70 ± 14.16	297 (208/89)	215/82	II-IVa	159	CT
Liao (29)	2021	China	Guangxi Medical University Cancer Hospital	January 2015 to June 2018	R	43.7 ± 11.0	286 (200/86)	209/77	III-IVa	220	MRI
Wang (21)	2023	China	Sichuan Cancer Hospital & Institute	July 2017 to August 2021	R	48.51 ± 11.32	184 (132/52)	134/50	II-IV	102	MRI
Hu (16)	2021	China	Fujian Cancer Hospital	January 2014 to July 2015	R	47.07 ± 11.19	284 (200/84)	220/64	III-IVa	172	MRI
Chen (45)	2024	China	General Hospital of Ningxia Medical University	December 2015 to April 2022	R	52.5(45.0-59.0)^*^	168 (114/54)	100/68	II-IV	98	MRI

Except where indicated, the data are the numbers of patients or means ± SDs; ^*^ Data are the medians, with IQRs in parentheses; R = retrospective; TP group, nasopharyngeal carcinoma treated with gemcitabine plus cisplatin; GP group, nasopharyngeal carcinoma treated with docetaxel plus cisplatin.

### 3.3 Image analyses

Detailed information on the image analyses included in the study and the predictive performance measures of the models are summarized in **Table 2** and **Supplementary Table S2**. With respect to the selection of the region of interest (ROI), the primary and lymph node gross tumor volume (GTV) was segmented in one study (17), whole tumors were segmented in seven studies (16, 18, 21, 27, 29, 31, 45), and only the largest axial slice was segmented in two studies (19, 20). The number of image features selected for the prediction models analyzed in this study ranged from 2 to 24. Except for one study that did not report model validation details (20), internal validation was performed in the remaining studies.

**Table 2:** Methodological characteristics and predictive performance of included radiomics models.

First author	Year	Segmentation method	Feature extraction software	Feature selection	Algorithm	Number of image features	Validation type​	Validation method	Validation sample size	Prediction model	SE	SP	ACC	AUC
Piao (20)	2021	Largest axial slice	AI Kit	ANOVA/MW test; Correlation analysis; LASSO	Logistic regression	2 radiomics features	Internal validation	NR	108	MRI Radiomics	0.857	0.833	0.843	0.905
Wang (17)	2022	Whole tumor involved lymph nodes	MATLAB	Logistic regression	Logistic regression	6 radiomics features	External validation	Independent testing	80	MRI Radiomics	0.783	0.825	0.813	0.925
Wang (19)	2018	Largest axial slice	MATLAB	LASSO	(LASSO) logistic regression	15 radiomics features	Internal validation	Bootstrap (1000 resamples)	120	MRI Radiomics	0.980	0.529	0.717	0.822
Zhang (18)	2020	Whole tumor	AI Kit	mRMR; LASSO	Logistic regression	20 radiomics features	External validation	Independent testing	34 (GP group)	MRI Radiomics	0.943	0.706	0.824	0.886
Zhao (27)	2020	Whole tumor	MATLAB	Two–sample t test; Logistic regression; LASSO	SVM	19 radiomics features	External validation	Independent testing	23	MRI SVM	0.882	0.833	0.870	0.873
										MRI SVM-Clinical	1.000	0.333	0.826	0.863
Yang (31)	2022	Whole tumor (SVM); 3 consecutive axial slices (DL)	PyRadiomics (SVM); ResNet50 (DL)	Univariate analysis; Recursive feature addition; ICC; Global Max Pooling	SVM or/and CNN	8 radiomics features and 7 DL features	External validation	Independent testing	89	CT CNN	0.881	0.617	0.742	0.811
										CT SVM	0.667	0.553	0.607	0.663
										CT SVM-Clinical	0.571	0.702	0.640	0.690
										CT SVM-CNN	0.524	0.702	0.618	0.694
Liao (29)	2021	Whole tumor	PyRadiomics and FCNN	Student’s t test or Mann–Whitney U test; Shapiro–Wilk and Levene’s Test; (LASSO)–logistic regression	Logistic regression or FCNN	24 radiomics features	External validation	Independent testing	86	MRI Radiomics	0.900	0.470	0.570	0.698
										MRI Radiomics-Clinical	0.900	0.545	0.628	0.755
						MRI FCNN				1.000	0.758	0.814	0.897
Wang (21)	2023	Whole tumor	PyRadiomics	mRMR; LASSO	Logistic regression	6 radiomics features	External validation	Independent testing	52	MRI Radiomics-Clinical	0.762	0.645	0.692	0.780
										MRI Radiomics	0.952	0.742	0.827	0.952
Hu (16)	2021	Whole tumor	AI Kit	mRMR; LASSO	Logistic regression	18 radiomics features	External validation	Independent testing	84	MRI Radiomics	0.788	0.588	0.667	0.770
Chen (45)	2024	Whole tumor	PyRadiomics	Student’s t test or Mann–Whitney U Test; LASSO	Logistic regression	14 radiomics features	External validation	Independent testing	54	MRI Radiomics	0.682	0.875	0.796	0.837
										MRI Radiomics-Clinical	0.818	0.938	0.889	0.901

GTV, gross tumor volume; DL, deep learning; LASSO, least absolute shrinkage and selection operator; mRMR, maximum relevance minimum redundancy; SVM, support vector machine; FCNN, fully connected neural network; CNN, convolutional neural network; SE, sensitivity; SP, specificity; ACC, accuracy. GP group, nasopharyngeal carcinoma treated with docetaxel plus cisplatin. NR, Not Reported.

### 3.4 Quality assessment

**Figure 2:**
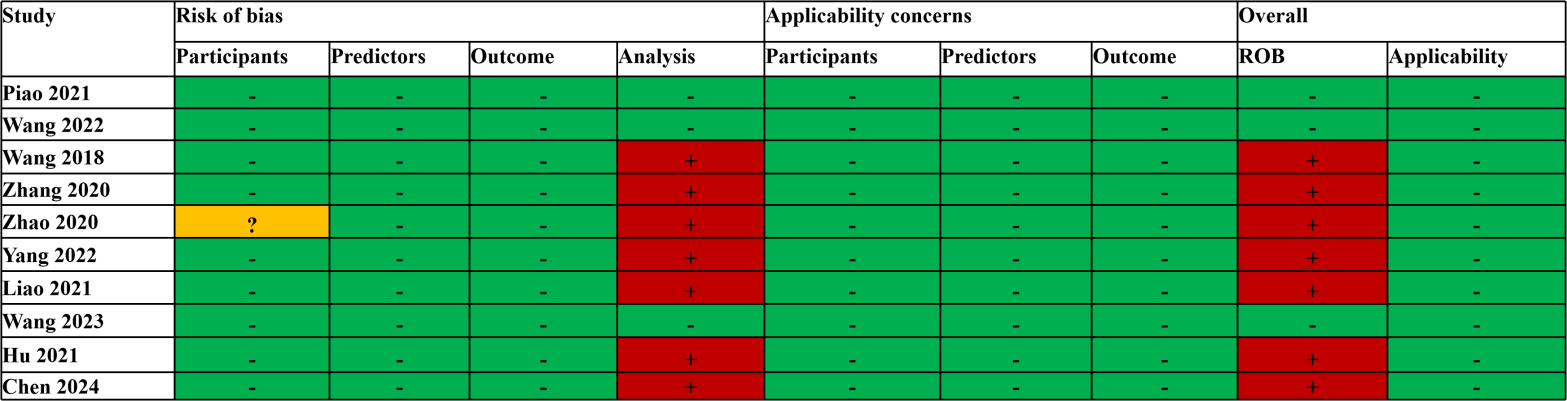
PROBAST results of the included studies. +, high; -, low;?, unclear.

The PROBAST assessment revealed that the overall risk of bias (ROB) was low in three studies (17, 20, 21). In the analysis, the number of EPV was less than 10 in six studies (16, 18, 19, 21, 29, 45), and one study (31) selected predictors based on univariate analysis, which had a high risk of bias; thus, the overall risk of bias was high. Another study (27) revealed that, among participants, the inclusion and exclusion criteria for all participants were vague; thus, the risk of bias was unclear, and the EPV in the analysis was less than 10, resulting in an overall high risk of bias. The overall applicability of all the studies’ concerns was low (**Figure 2**). The average RQS for the 10 studies was 16.2 (45.0%). The pre-processing steps were carried out following the IBSI guidelines, with an overall adherence rate of 60.0% (42/70). Detailed evaluations are available in **Supplementary Material** (**Supplementary Tables S3**, **S4**, **S5**).

### 3.5 Traditional meta-analysis

**Figure 3:**
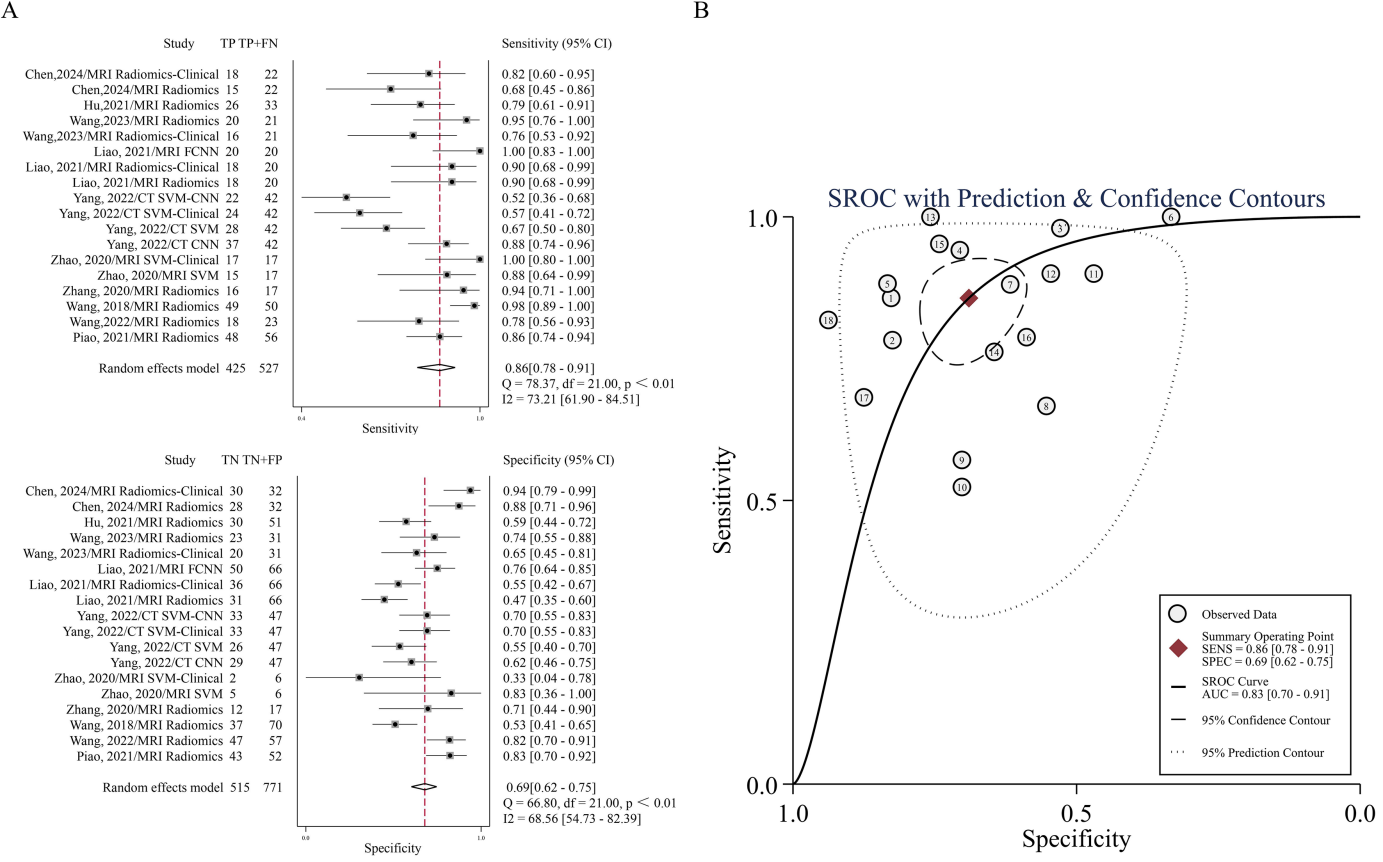
**(A)** Forest plots of the pooled sensitivity and specificity. **(B)** SROC curve for the ability of radiomics to predict IC efficacy in NPC patients.

In the 18 cohorts of 10 studies in which radiomics was used to predict the efficacy of IC in NPC, the pooled sensitivity and specificity were 0.86 (95% CI: 0.78-0.91) and 0.69 (95% CI: 0.62-0.75), respectively. From the plotted SROC curve, an AUC of 0.83 (95% CI: 0.70-0.91) was obtained. The forest plot of the sensitivity and specificity of the predictive performance of the radiomics model is shown in **Figure 3A**, and the SROC curve is shown in **Figure 3B**.

Methodologically, this study computed pooled HSROC AUC for individual models only when two or more studies were available. The MRI radiomics model (8 studies (16–21, 29, 45)) demonstrated pooled sensitivity of 0.88 (95% CI: 0.80–0.90), specificity of 0.70 (95% CI: 0.59–0.80), and HSROC AUC of 0.88 (95% CI: 0.84–0.90). Similarly, the MRI radiomics-clinical model (3 studies (21, 29, 45)) showed pooled sensitivity of 0.83 (95% CI: 0.71–0.90), specificity of 0.74 (95% CI: 0.47–0.90), and HSROC AUC of 0.84 (95% CI: 0.81–0.87). Detailed performance metrics are presented in **Supplementary Material** (**Supplementary Figure S1**). Models with fewer than two studies were excluded due to insufficient meta-analytic feasibility.

### 3.6 Heterogeneity exploration and meta-regression

The *I*^2^ statistic reveals significant heterogeneity in sensitivity (*I*^2^ = 78.31%) and moderate heterogeneity in specificity (*I*^2^ = 74.55%) (**Supplementary Table S6**). As shown in **Supplementary Tables S7**, eight covariates were used to explore potential sources of heterogeneity. Meta-regression and joint model analysis indicated the following factors as contributors to significant heterogeneity in the meta-analysis: device type (MRI vs. CT) (*P* < 0.01), sample (≥200 vs. <200) (*P =* 0.02). The device type was a highly heterogeneous source (*I*^2^ = 82.00%), while the CT equipment came from the same study. We therefore performed a subgroup analysis of multiple studies of MRI devices.

Results were analyzed in subgroups according to MRI device (**Table 3**). Compared to studies with an EPV <10, those with EPV≥10 showed lower pooled sensitivity (0.85 vs. 0.90, *P =* 0.01) but higher specificity (0.77 vs. 0.67, *P =* 0.68, nonsignificant). The sensitivity of 3D ROI was lower than that of 2D ROI (0.87 vs. 0.93, *P =* 0.03), and the specificity of 3D ROI was similar (0.71 vs. 0.70, *P =* 0.59, nonsignificant). Studies that validated the predictive performance of the model on external validation cohorts manifested lower sensitivity (0.87 vs. 0.93, *P =* 0.03) and similar specificity (0.71 vs. 0.70, *P =* 0.59, nonsignificant). Linear models demonstrated lower sensitivity than nonlinear models (0.87 vs. 0.97, *P =* 0.03) and similar specificity (0.71 vs. 0.70, *P =* 0.58, nonsignificant). Compared to using ≥10 features, those with < 10 features showed lower pooled sensitivity (0.85 vs. 0.90, *P =* 0.28, nonsignificant) but higher specificity (0.77 vs. 0.67, *P =* 0.04). Higher RQS correlated with decreased sensitivity (0.88 vs. 0.91, *P =* 0.02) and specificity (0.69 vs. 0.74, *P =* 0.15, nonsignificant). Notably, studies with larger sample sizes (≥200) showed higher pooled sensitivity (0.90 vs. 0.88, *P =* 0.15, nonsignificant) but lower specificity(0.61 vs. 0.76, *P =* 0.01) compared to smaller cohorts.

**Table 3:** Investigation of heterogeneity using meta-regression and subgroup analysis of MRI devices.

Parameter	Category	N	Sensitivity	*P_1_*	Specificity	*P_2_*	Joint model analysis		
							*P*	*I²*	LRT chi^2^
EPV	≥10	4	0.85 (0.75–0.96)	**0.01**	0.77 (0.64–0.90)	0.68	0.49	0.0 (0.0–100.0)	1.45
	<10	10	0.90 (0.85–0.96)		0.67 (0.57–0.78)				
ROI	3D	12	0.87 (0.81–0.93)	**0.03**	0.71 (0.61–0.80)	0.59	0.51	0.0 (0.0–100.0)	1.35
	2D	2	0.93 (0.86–1.00)		0.70 (0.47–0.92)				
Validation	External validation	12	0.87 (0.81–0.93)	**0.03**	0.71 (0.61–0.80)	0.59	0.51	0.0 (0.0–100.0)	1.35
	Others	2	0.93 (0.86–1.00)		0.70 (0.47–0.92)				
Algorithms	Linear	11	0.87 (0.81–0.92)	**0.03**	0.71 (0.62–0.81)	0.58	0.11	55.0 (0.0–100.0)	4.44
	Nonlinear	3	0.97 (0.92–1.00)		0.70 (0.48–0.93)				
Feature	≥10	10	0.90 (0.85–0.96)	0.28	0.67 (0.57–0.78)	**0.04**	0.49	0.0 (0.0–100.0)	1.45
	<10	4	0.85 (0.75–0.96)		0.77 (0.64–0.90)				
RQS	≥16.2	10	0.88 (0.81–0.94)	**0.02**	0.69 (0.58–0.80)	0.15	0.56	0.0 (0.0–100.0)	1.15
	<16.2	4	0.91 (0.83–0.98)		0.74 (0.59–0.88)				
Sample	≥200	5	0.90 (0.83–0.98)	0.15	0.61 (0.47–0.75)	**0.01**	0.22	35.0 (0.0–100.0)	3.07
	<200	9	0.88 (0.82–0.94)		0.76 (0.67–0.85)				

EPV, events per variable; ROI, region of interest; RQS, radiomics quality score.

The bold values denote P<0.05.

### 3.7 Network evidence diagram

**Figure 4:**
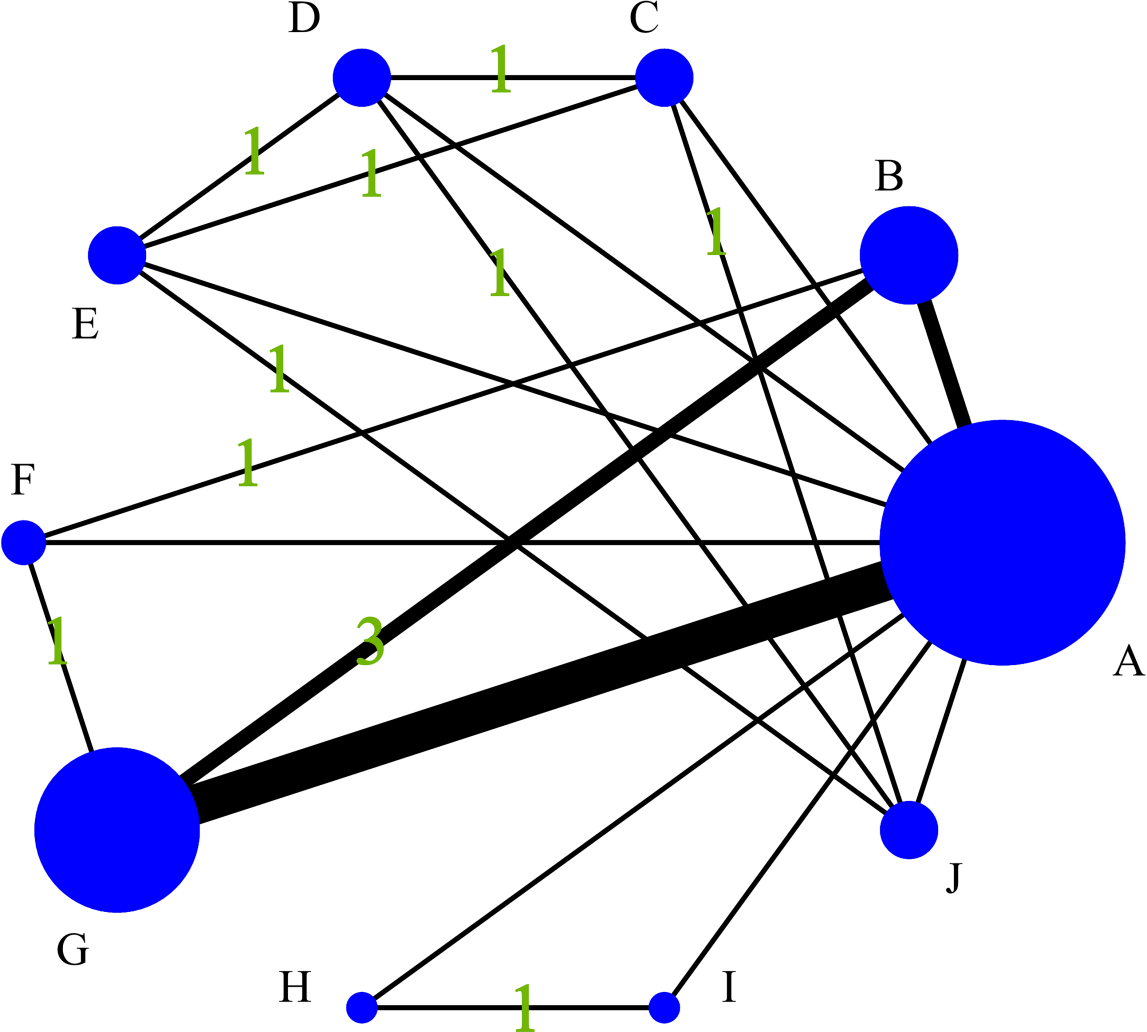
Diagram of the evidence network included in this study. **(A)**, Control group (clinical gold standard for IC efficacy assessment based on RECIST 1.1); **(B)**, MRI Radiomics-Clinical model; **(C)**, CT CNN model; **(D)**, CT SVM model; **(E)**, CT SVM-Clinical model; **(F)**, MRI FCNN model; **(G)**, MRI Radiomics model; **(H)**, MRI SVM model; **(I)**, MRI SVM-Clinical model; **(J)**, CT SVM-CNN model.

**Figure 4** quantifies the density of direct comparative evidence within the NMA, demonstrating that both the MRI Radiomics model and MRI Radiomics-Clinical model have undergone extensive validation across multiple studies.

### 3.8 Consistency and inconsistency analysis

The sensitivity, specificity, and accuracy of all included studies were analyzed via inconsistency analysis employing the node-splitting method, and the results indicated consistency among the direct and indirect evidence of all outcomes (all *p* > 0.05). Therefore, the consistency model was applied in the current study.

### 3.9 Network meta-analysis

NMA revealed that the CT CNN model was superior to the CT SVM model (OR = 3.47, 95% CI: 1.16-10.38), the CT SVM-Clinical model (OR = 5.15, 95% CI: 1.75-15.15), and the CT SVM-CNN model (OR = 6.21, 95% CI: 2.12-18.23) in sensitivity. The MRI FCNN model was superior to the MRI Radiomics-Clinical model (OR = 2.71, 95% CI: 1.34-5.48) and the MRI Radiomics model (OR = 3.14, 95% CI: 1.55-6.33) in specificity. The MRI FCNN model was superior to the MRI Radiomics-Clinical model (OR = 2.72, 95% CI: 1.13-6.60) and the MRI Radiomics model (OR = 2.90, 95% CI: 1.20-6.99) in accuracy. The league table of the outcome indicators is shown in **Supplementary Material Table S6**.

### 3.10 SUCRA values

**Figure 5:**
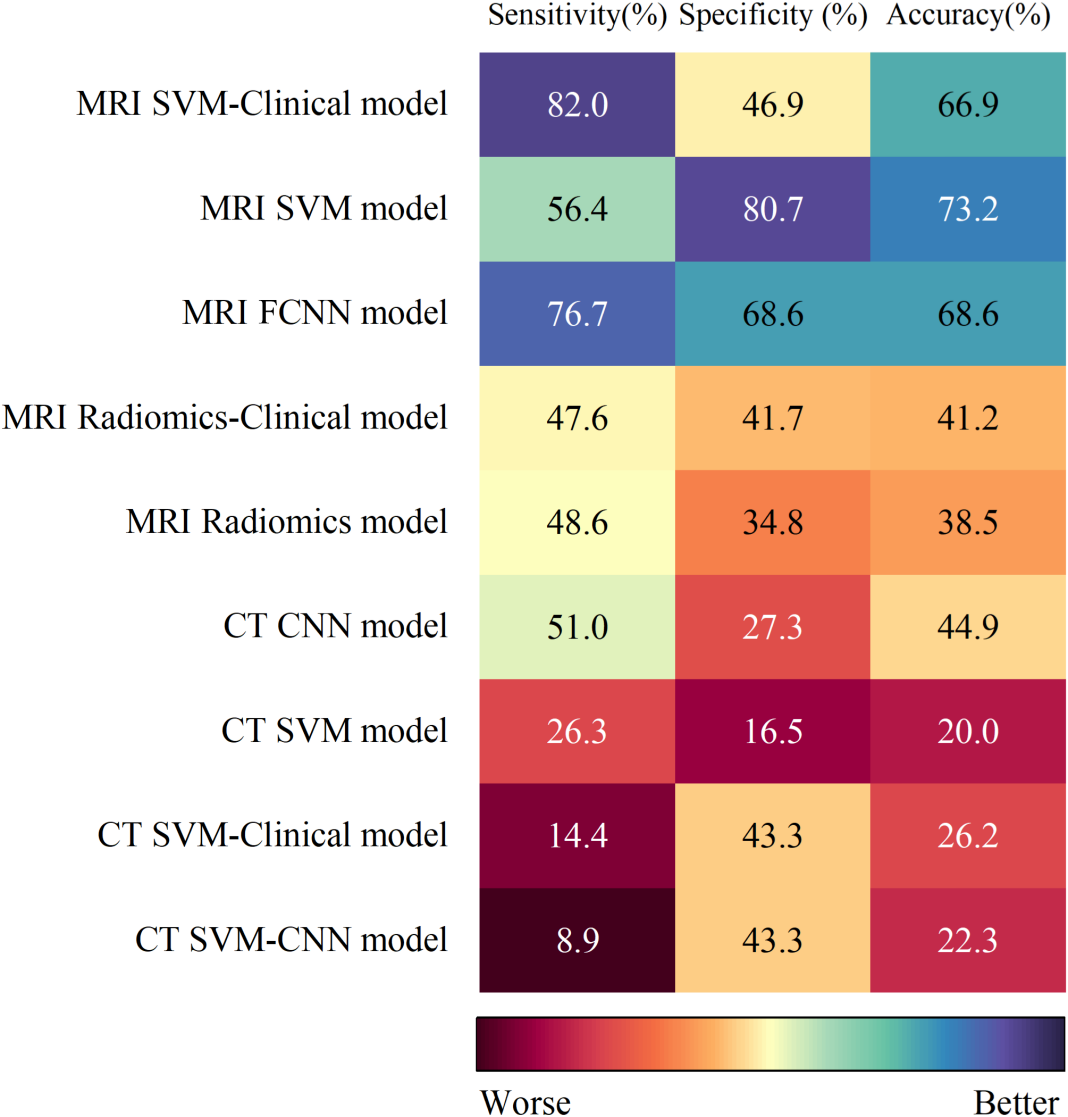
The SUCRA values for the 9 prediction models with 3 endpoints.

**Figure 6:**
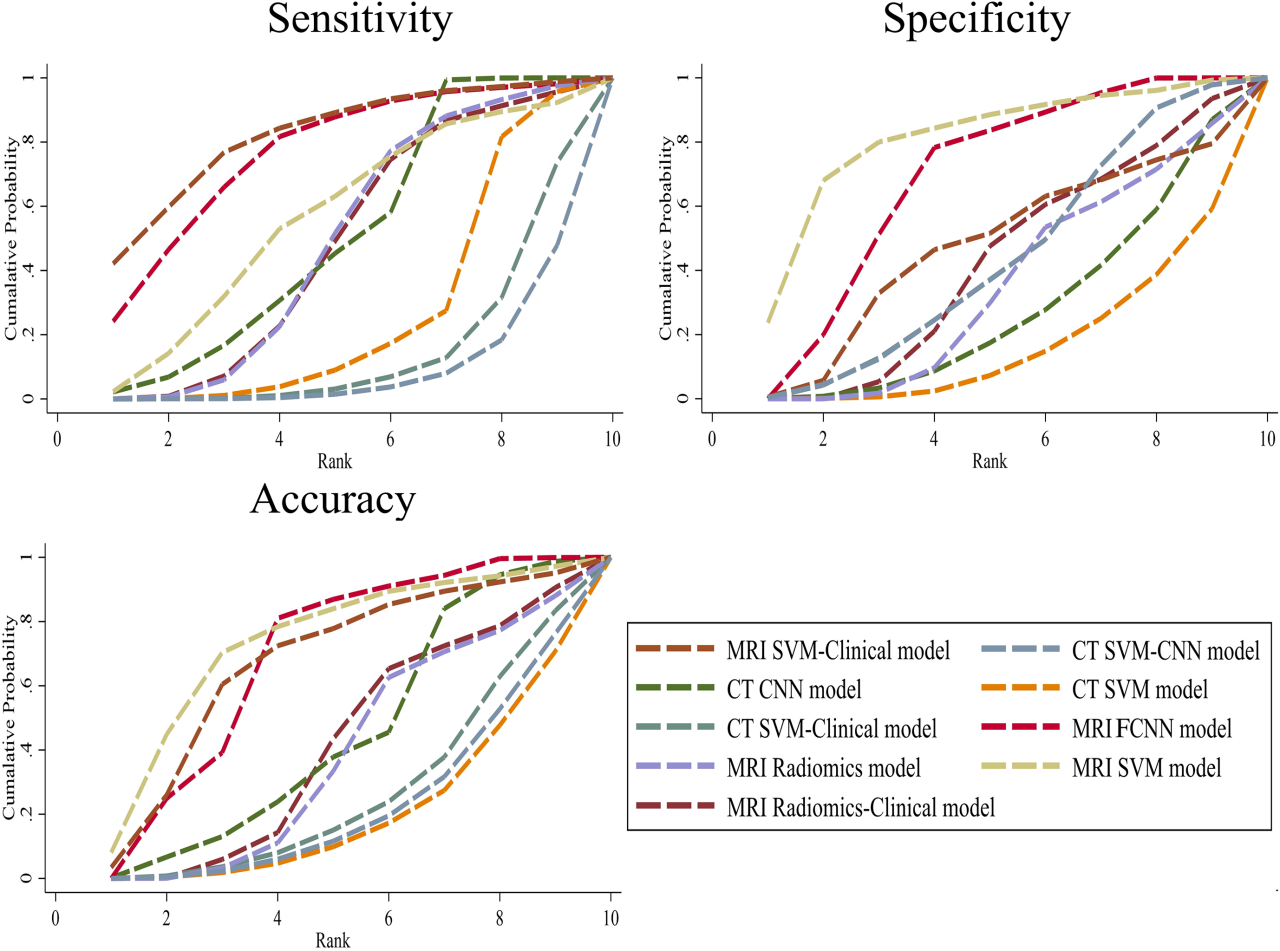
Cumulative ranking probability plots for the 9 prediction models with 3 endpoints.

The SUCRA values for the 9 prediction models are summarized in **Figure 5**. The MRI SVM model had the highest specificity, and accuracy (SUCRA) at 80.7%, and 73.2%, respectively. The MRI SVM-Clinical model had the highest sensitivity (82.0%). The sensitivity, specificity, and accuracy of the MRI FCNN model ranked the top two, which were 76.7%, 68.6%, and 68.6%, respectively. Among the CT methods, the CNN model of DL had the highest SUCRA values for sensitivity, and accuracy at 51.0%, and 44.9%, respectively. The SUCRA curves are presented in **Figure 6**.

### 3.11 Sensitivity analyses

**Figure 7:**
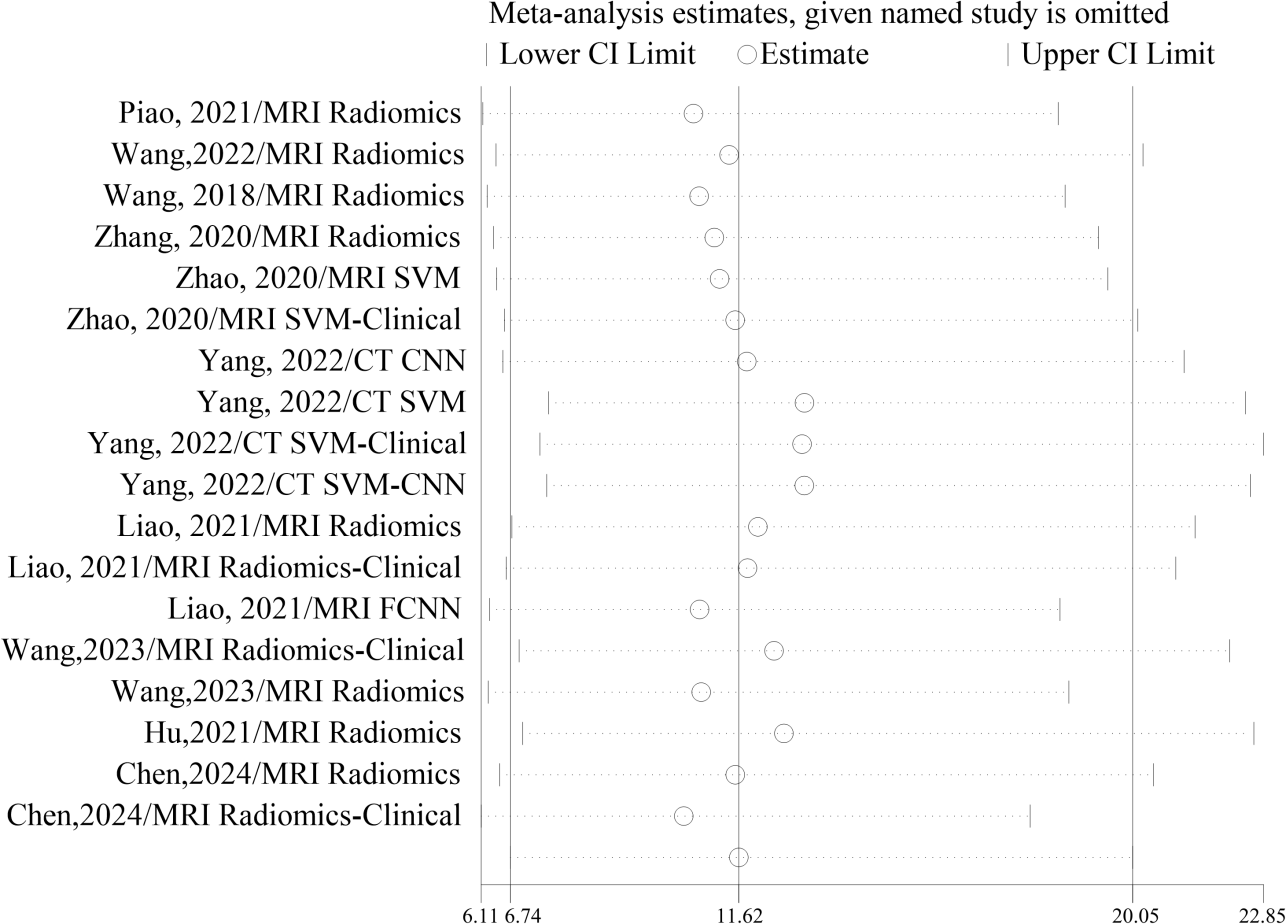
Results of sensitivity analyses.

No significant changes were observed when each included study was eliminated from the analysis one by one. The results of sensitivity analyses for each study are shown in **Figure 7**.

### 3.12 Assessment of publication bias

**Figure 8:**
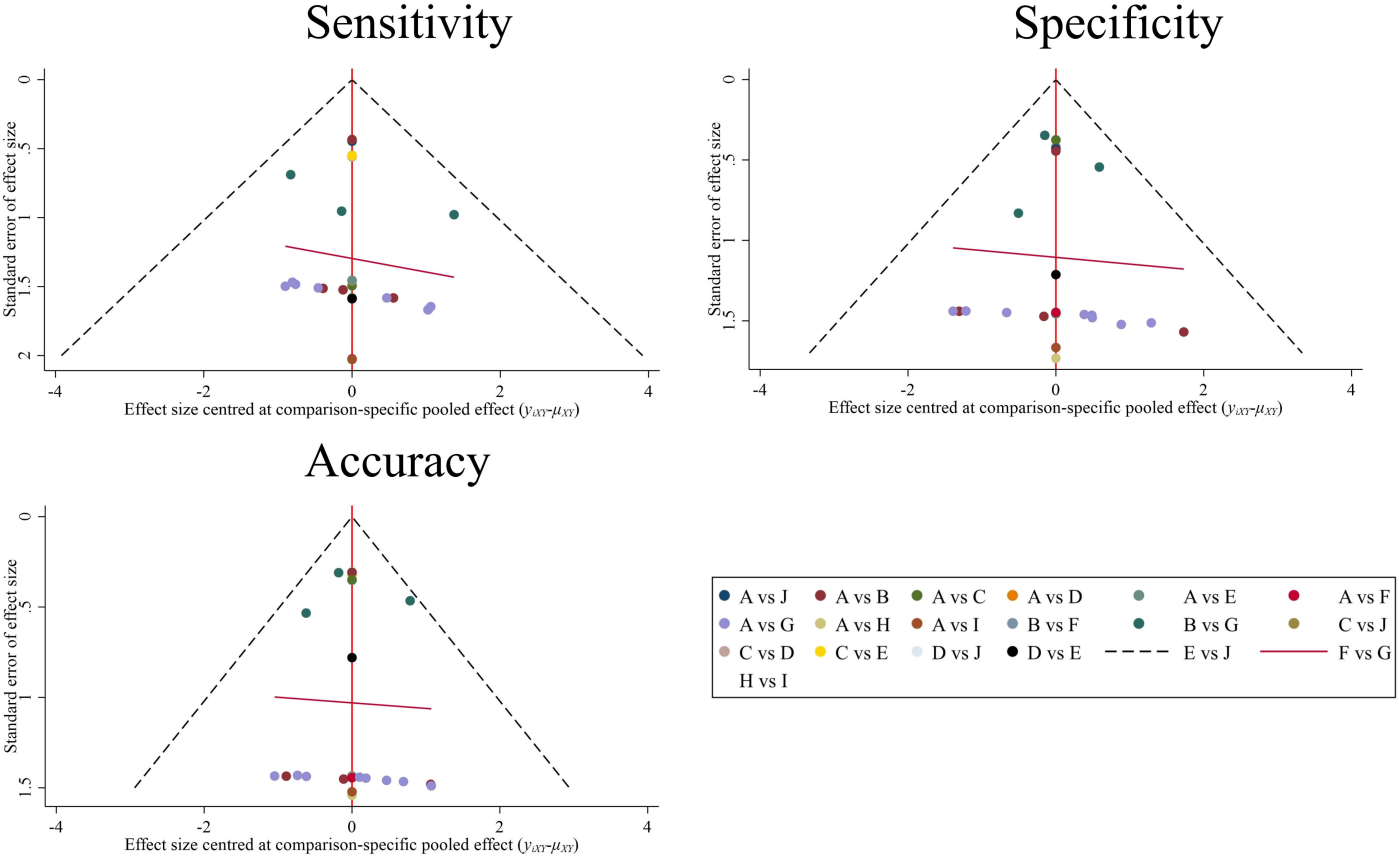
Comparison-adjusted funnel plot for 9 prediction models. Each data point represents a single study. **(A)**, Control group (clinical gold standard for IC efficacy assessment based on RECIST 1.1). **(B)**, MRI Radiomics-Clinical model; **(C)**, CT CNN model; **(D)**, CT SVM model; **(E)**, CT SVM-Clinical model; **(F)**, MRI FCNN model; **(G)**, MRI Radiomics model; **(H)**, MRI SVM model; **(I)**, MRI SVM-Clinical model; **(J)**, CT SVM-CNN model.

The comparison-adjusted funnel plots (**Figure 8**) show roughly symmetrical scatter points of the same color, indicating the negligible presence of publication bias or other forms of bias within the studies, as confirmed by Deek’s test (*P* = 0.427 > 0.05). This symmetry bolstered the reliability of the findings.

## 4 Discussion

The efficacy of IC serves as a robust predictor of survival outcomes in NPC patients post-IC (31). Prior evidence indicates a CR/PR rate of 76.9% following IC in NPC cohorts (46), underscoring that not all patients derive clinical benefit from IC. Our study demonstrated that the MRI SVM model achieved the highest specificity (80.7%), effectively reducing FP and ensuring aggressive therapies like IC are reserved for high-confidence responders. Conversely, the MRI SVM-Clinical model exhibited peak sensitivity (82.0%), capturing more true responders at the cost of increased over-treatment and potential exposure to IC-associated toxicities without survival benefit. In resource-limited settings, model thresholds can be calibrated to prioritize cost-effectiveness (e.g., avoiding IC in low-response-probability subgroups). For high-toxicity regimens such as cisplatin-based IC (47), stringent thresholds may be preferred to mitigate harm. Iterative adjustments based on real-world outcome monitoring (e.g., post-IC surveillance) further enable dynamic refinement of decision boundaries as evidence evolves.

Both our study and Yang et al. (31) focused on assessing radiomics to evaluate IC efficacy in NPC. Notably, compared with Yang et al. (31), our research included more primary studies and models (6 vs. 10 primary studies and 6 vs. 18 models), compared multiple radiomics algorithms, standardized the definitions of TP/FP cases to clarify model performance, and applied meta-regression and subgroup analyses to address heterogeneity. We performed the first NMA to compare the value of 9 different models in predicting IC efficacy in NPC. Based on the SUCRA values of different models, the following conclusions are drawn: Radiomics based on MRI and CT serves as a viable exploratory tool for predicting IC efficacy in NPC. Among them, the MRI FCNN model ranked in the top two for sensitivity, specificity, and accuracy, indicating superior overall predictive performance. The MRI SVM model had better specificity and accuracy, while the MRI SVM-Clinical model had better sensitivity. The above nonlinear ML combined with radiomics had a good predictive performance for the noninvasive identification of IC treatment response in NPC. Moreover, Li et al. (48) reported that the FCNN model exhibited optimal performance in evaluating HER2-low breast cancer undergoing neoadjuvant therapy. Therefore, the combination of FCNN and radiomics may be a promising method for predicting NPC treatment response.

The three studies (16, 29, 45) in this NMA showed that the MRI Radiomics-Clinical model could improve the predictive performance over that of the MRI Radiomics model. However, Wang et al. (21) showed that the MRI Radiomics-Clinical model could not improve predictive performance. Our research revealed that, compared with the MRI Radiomics model, the MRI Radiomics-Clinical model had greater specificity, and a lower sensitivity in the surca ranking. This is consistent with the pooled sensitivity and specificity ranking, which confirms the surca value accuracy of NMA. Owing to the lack of data, the clinical characteristics (such as EBV-DNA, LDH, etc.) that were independent predictors in other studies were not included in the study of Wang et al. (21), which might explain why the performance of the MRI Radiomics-Clinical model was lower than that of the MRI Radiomics model. In future studies, more relevant clinical data should be included to analyze the correlation between tumor microenvironment and radiomics features.

Notably, the *I*^2^ values of specificity, and accuracy of the 9 models ranged were 74.55% and 69.51%, respectively, indicating moderate heterogeneity, whereas the *I*^2^ value of sensitivity was 78.31%, greater than 75%, indicating high heterogeneity. Device type (MRI vs. CT)(*P* < 0.01) was the source of high heterogeneity (**Supplementary Table S7**). MRI led the studies (9/10), while CT was the only study (**Table 1**). The prediction efficiency of the MRI models was generally greater than that of the CT models. The possible reason is that MRI has significantly better resolution of soft tissue than CT does and effectively shows the range of the parapharyngeal space, skull base, and intracranial tumors (49), thus providing more realistic internal characteristics of the tumors. In the model based on CT images, we found that the SUCRA values of sensitivity, and accuracy of the CNN model were the highest, and sensitivity, and accuracy were even higher than those of the linear algorithms MRI models (MRI Radiomics model and MRI Radiomics-Clinical model) (**Figure 5**). The CNN has multiple layers of neuron-like computational connections, which sets a target-size bounding box on the lesion area, evaluates the malignant probability of the identified lesion, and achieves end-to-end output of entire image sequences (50). It reduces the need for manual intervention and traditional preprocessing steps by automating the extraction of complex features. Comes et al. (51) showed that MRI-based radiomics combined with a CNN model could predict the pathological CR of patients with breast cancer to neoadjuvant chemotherapy early, with an AUC of 0.82 (95% CI: 0.75-0.88). Therefore, the combination of CNN and MRI in future studies of NPC can provide a new way to predict the efficacy of IC.

In MRI-based radiomics subgroup analyses, studies with EPV ≥ 10 demonstrated significantly lower sensitivity than EPV <10 cohorts (0.85 vs. 0.90, *P =* 0.01). This inverse relationship indicates that low EPV (<10) may induce model overfitting to training data noise, thereby inflating sensitivity estimates in smaller cohorts (52). For ROI segmentation, 3D showed reduced sensitivity versus 2D (0.87 vs. 0.93, *P =* 0.03). While 2D segmentation risks missing heterogeneous tumor regions (increasing FN), 3D segmentation introduces non-target biological signals (e.g., necrosis/edema) (53). This sensitivity reduction reflects technical-biological complexity coupling, not methodological inferiority. The future requires dynamically optimizing ROI and adaptive segmentation based on necrosis proportion (54), coupled with analyzing how tumor microenvironment components (e.g., necrotic core vs. active margins) influence IC sensitivity, to unlock the potential of 3D imaging in resolving spatial heterogeneity of treatment effects.

External validation cohorts exhibited lower sensitivity than internal cohorts (0.87 vs. 0.93, *P =* 0.03), indicating overfitting risks. Large-sample studies (≥200 patients) showed poorer specificity than small-sample cohorts (0.61 vs. 0.76, *P =* 0.01), likely due to increased patient diversity compromising model discriminability. Conversely, small samples risk overfitting (e.g., Zhao et al. (27), validation n = 23, sensitivity = 100%). Therefore, on the basis of expanding the sample size, punitive modeling should be promoted simultaneously to solve the risk of overfitting in small samples. Nonlinear algorithms outperformed linear algorithms in sensitivity (0.97 vs. 0.87, *P =* 0.03), aligning with NPC’s complex response dynamics (55) and SUCRA rankings (**Figure 5**). Models with <10 features achieved higher specificity than those with ≥10 features (0.77 vs. 0.67, *P =* 0.04). This finding demonstrates that reduced feature quantity can mitigate model complexity, thereby decreasing false-positive predictions and enhancing both the robustness and diagnostic accuracy of radiomics models in predicting IC efficacy in NPC. While a previous study reported an average RQS of 31.0% (56), our analysis showed that the average RQS increased to 45.0%, which indicates a methodological advance. However, Low RQS studies correlated with inflated sensitivity (0.88 vs. 0.91, *P =* 0.02), highlighting uncorrected optimism bias. Therefore, we advocate that the methodological radiomics score (METRICS) be followed in future studies, a new scoring tool for assessing the methodological quality of the radiomics research (57). The “feature stability” and “model development” domains of METRICS systematically identify optimism bias sources overlooked by RQS, while its core domains of “clinical utility” and “reproducibility” address clinically detached optimism bias at its root. According to **Supplementary Table S5**, only 40% of studies (4/10) adhered to IBSI preprocessing protocols, with critical gaps in gray-level discretization (40% implementation) and image interpolation (40%), collectively contributing to diminished feature stability.

There were several limitations in this study. First, studies with two arms or more were relatively rare, and the extractable data were limited, without forming a closed loop. Second, the ROB ratings of some studies included in this study were high, mainly because the EPV was less than 10, which may have led to an increased risk of overfitting the prediction model. Future model development studies must ensure EPV ≥10 via *a priori* sample size estimation based on radiomics-specific guidelines. Third, the universal absence of external validation fundamentally limits the generalizability of current radiomics models, which prevents robust validation across institutionally heterogeneous cohorts.

## 5 Conclusions

This study provides a clinical reference for IC efficacy prediction in NPC by synthesizing radiomics evidence. As an exploratory tool, radiomics demonstrates potential; nevertheless, its performance generalizability requires cautious interpretation owing to technical heterogeneity. MRI-based nonlinear models and clinical-integrated frameworks demonstrate significant potential for clinical translation; however, achieving reliable deployment necessitates three critical steps: (1) standardized protocols adhering to IBSI/METRICS/RQS guidelines to reduce heterogeneity, (2) rigorous validation frameworks employing prospective multicenter designs to address generalization gaps, and (3) biological mechanism exploration linking imaging features to tumor microenvironment dynamics. Collectively, these strategies will facilitate the transition of radiomics from technical exploration to clinical utility in NPC precision oncology.

## Data Availability

The original contributions presented in the study are included in the article/**Supplementary Material**. Further inquiries can be directed to the corresponding author.
