# Supplementary material for: Predictive performance of MRI and CT radiomics in predicting the response to induction chemotherapy in nasopharyngeal carcinoma: a network meta-analysis
Source: Front Oncol. 2025 Nov 13;15:1590420. doi: 10.3389/fonc.2025.1590420 (PMC12657194; doi:10.3389/fonc.2025.1590420)
Supplement: Supplementary file 1 [file DataSheet1.docx]

Supplementary Material

**Table S1** Detailed search strategy.

**Table S2** Summary of details of the image analyses.

**Table S3** PROBAST Quality Analysis Results.

**Table S4** RQS of individual studies.

**Table S5** Adherence rate of IBSI pre-processing steps.

**Table S6** League table of the five outcome indicators.

**Table S7** Investigation of heterogeneity using meta-regression.

**Figuer S1** Single model predictive performance (SROC curve).

**Table S1** Detailed search strategy.

| **Database：PubMed** | **Date Range：Inception to May 25, 2024** | **Language Restrictions：English** |
| --- | --- | --- |
| #1 search "nasopharyngeal carcinoma"[MeSH Terms] OR ("nasopharyngeal"[All Fields] AND "carcinoma"[All Fields]) OR "nasopharyngeal carcinoma"[All Fields] OR ("nasopharyngeal carcinoma"[MeSH Terms] OR ("nasopharyngeal"[All Fields] AND "carcinoma"[All Fields]) OR "nasopharyngeal carcinoma"[All Fields] OR ("carcinoma"[All Fields] AND "nasopharyngeal"[All Fields]) OR "carcinoma nasopharyngeal"[All Fields]) OR ("nasopharyngeal carcinoma"[MeSH Terms] OR ("nasopharyngeal"[All Fields] AND "carcinoma"[All Fields]) OR "nasopharyngeal carcinoma"[All Fields] OR ("carcinomas"[All Fields] AND "nasopharyngeal"[All Fields]) OR "carcinomas nasopharyngeal"[All Fields]) OR ("nasopharyngeal carcinoma"[MeSH Terms] OR ("nasopharyngeal"[All Fields] AND "carcinoma"[All Fields]) OR "nasopharyngeal carcinoma"[All Fields] OR ("nasopharyngeal"[All Fields] AND "carcinomas"[All Fields]) OR "nasopharyngeal carcinomas"[All Fields])  #2 search (Radiomic) OR "Radiomics"[Title/Abstract]  #3 search (((Machine Learning[Title/Abstract]) OR (Learning, Machine[Title/Abstract])) OR (Hierarchical Learning[Title/Abstract])) OR (Learning, Hierarchical[Title/Abstract])  #4 search (((Deep Learning[Title/Abstract]) OR (Learning, Deep[Title/Abstract])) OR (Hierarchical Learning[Title/Abstract])) OR (Learning, Hierarchical[Title/Abstract])  #5 search #2 or #3 or #4  #6 search #1 and #5 | | |
| **Database：Cochrane library** | **Date Range：Inception to May 25, 2024** | **Language Restrictions：English** |
| #1 search (Nasopharyngeal Carcinoma):ti,ab,kw OR (Carcinoma, Nasopharyngeal):ti,ab,kw OR (Carcinomas, Nasopharyngeal):ti,ab,kw OR (Nasopharyngeal Carcinomas):ti,ab,kw  #2 search (Radiomic):ti,ab,kw OR (Radiomics):ti,ab,kw  #3 search (Machine Learning):ti,ab,kw OR (Learning, Machine):ti,ab,kw OR (Hierarchical Learning):ti,ab,kw OR (Learning, Hierarchical):ti,ab,kw  #4 search (Deep Learning):ti,ab,kw OR (Learning, Deep):ti,ab,kw OR (Hierarchical Learning):ti,ab,kw OR (Learning, Hierarchical):ti,ab,kw  #5 search #2 or #3 or #4  #6 search #1 and #5 | | |
| **Database：Web of science** | **Date Range：Inception to May 25, 2024** | **Language Restrictions：English** |
| Carcinomas, Nasopharyngeal OR Nasopharyngeal Carcinomas)  #2 search TS= (Radiomic OR Radiomics)  #3 search TS= (Machine Learning OR Learning, Machine OR Hierarchical Learning OR Learning, Hierarchical)  #4 search TS= (Deep Learning OR Learning, Deep OR Hierarchical Learning OR Learning, Hierarchical)  #5 search #2 or #3 or #4  #6 search #1 and #5 | | |
| **Database：Embase** | **Date Range：Inception to May 25, 2024** | **Language Restrictions：English** |
| #1 search 'carcinoma of the epipharynx'/exp OR 'carcinoma of the epipharynx' OR 'carcinoma of the nasopharynx'/exp OR 'carcinoma of the nasopharynx' OR 'carcinoma of the rhinopharynx'/exp OR 'carcinoma of the rhinopharynx' OR 'epipharyngeal carcinoma'/exp OR 'epipharyngeal carcinoma' OR 'epipharynx carcinoma'/exp OR 'epipharynx carcinoma' OR 'naso-pharyngeal carcinoma'/exp OR 'naso-pharyngeal carcinoma' OR 'nasopharyngeal carcinoma'/exp OR 'nasopharyngeal carcinoma' OR 'postnasal space carcinoma'/exp OR 'postnasal space carcinoma' OR 'rhino-pharyngeal carcinoma'/exp OR 'rhino-pharyngeal carcinoma' OR 'rhinopharyngeal carcinoma'/exp OR 'rhinopharyngeal carcinoma' OR 'rhinopharynx carcinoma'/exp OR 'rhinopharynx carcinoma' OR 'nasopharynx carcinoma'/exp OR 'nasopharynx carcinoma'  #2 search 'radiomic' OR 'radiomics'  #3 search 'machine learning' OR 'learning, machine' OR 'hierarchical learning' OR 'learning, hierarchical'  #4 search 'Deep Learning' OR 'Learning, Deep' OR 'Hierarchical Learning' OR 'Learning, Hierarchical'  #5 search #2 or #3 or #4  #6 search #1 and #5 | | |

| **First Author** | **Year** | **Segmentation Software** | **ROI** | **Sequence​** | **Segmentation Approach** | **Feature Processing​** | **Feature Extracted** | **Type of Features** | **Prediction Model** |
| --- | --- | --- | --- | --- | --- | --- | --- | --- | --- |
| Piao [20] | 2021 | ITK-SNAP | Primary tumor  only (lymph nodes excluded) | CE T1WI | Manual, 2D  Largest axial slice | Pre-extraction of features from the training set | First-order , Second-order (GLCM, GLGCM) and Wavelet features (396 features total). | Radiomics features | MRI Radiomics |
| Wang [17] | 2022 | MIM | Primary tumor  involved lymph nodes | T1WI, T2WI, and CE T1WI | Manual, 3D | Pre-extraction of features from the training set | 4 shape features volume. 3 types of First-orde. 4 types of Texture Features. Wavelet features (31920 features total). | Radiomics features | MRI Radiomics |
| Wang [19] | 2018 | ImageJ 1.50i | Primary tumor  only (lymph nodes excluded) | T1WI, T2WI, CE T1WI, and T2WI FS | Manual, 2D  Largest axial slice | Pre-extraction of features from the training set | First-order, GLCM, GLRLM, Gabor, and Wavelet Features (591 features total). | Radiomics features | MRI Radiomics |
| Zhang [18] | 2020 | ITK-SNAP | Primary tumor  only (lymph nodes excluded) | T1WI, T2WI, and CE T1WI | Manual, 3D | Pre-extraction of features from the training set | First-order, Form factor, GLCM, GLRLM, and GLSZM features (1188 features total). | Radiomics features | MRI Radiomics |
| Zhao [27] | 2020 | MITK | Primary tumor  only (lymph nodes excluded) | T1WI, T2WI, and CE T1WI | Manual, 3D | Pre-extraction of features from the training set | First-order , Texture , and Wavelet features (4503 features total). | Radiomics features  Radiomics and clinical features | MRI SVM  MRI SVM-Clinical |
| Yang [31] | 2022 | ITK-SNAP | Primary tumor  only (lymph nodes excluded) | CE-CT | SVM: Manual, 3D  DL: 3 consecutive slices with maximum cross-sectional area | SVM: Pre-extraction of features from the training set  End-to-end DL model ( FCNNs trained directly on image data) | Handcrafted Radiomics: First-order，Shape，and Second-order features (GLCM, GLRLM, GLSZM，GLDM, and NGTDM) (752 features total).  DL (ResNet50) 2048 features total. | Radiomics features  Radiomics and clinical features  DL features  Radiomics and DL features | CT SVM  CT SVM-Clinical  CT CNN  CT SVM-CNN |
| Liao [29] | 2021 | ITK-SNAP | Primary tumor  only (lymph nodes excluded) | T2WI and CE T1WI | Manual, 3D | Pre-extraction of features from the training set | Shape, First-order, Texture (GLCM, GLRLM, GLSZM, GLDM, and NGTDM), and Wavelet features (2074 features total). | Conventional radiomics features  Radiomics and clinical features  DL features | MRI Radiomics  MRI Radiomics-Clinical  MRI FCNN |
| Wang [21] | 2023 | ITK-SNAP | Primary tumor  only (lymph nodes excluded) | T2WI and CE T1WI | Manual, 3D | Pre-extraction of features from the training set | 4 Shape, 17 First-order, and 75 Texture features (212 features total). | Radiomics features  Radiomics and clinical features | MRI Radiomics  MRI Radiomics-Clinical |
| Hu [16] | 2021 | ITK-SNAP | Primary tumor  only (lymph nodes excluded) | T2WI and CE T1WI | Manual, 3D | Pre-extraction of features from the training set | Shape, First-order, GLCM, GLRLM, GLSZM, and Wavelet features (680 features total). | Radiomics features | MRI Radiomics |
| Chen [45] | 2024 | 3D Slicer | Primary tumor  only (lymph nodes excluded) | T1WI, CE T1WI, and T2WI FS | Manual, 3D | Pre-extraction of features from the training set | Shape, First-order, Texture, and Wavelet features (3111 features total). | Radiomics features | MRI Radiomics |

**Table S2** Summary of details of the image analyses

Note: DL, deep learning; SVM, support vector machine; FCNN, fully connected neural network; CNN, convolutional neural network.

**Table S3** PROBAST Quality Analysis Results

The PROBAST checklist is a tool that can be utilized for the critical appraisal of studies involved in developing, validating, or updating prediction models for individualized prediction. It comprises 20 signalling questions categorized into four domains: Participants, predictors, outcome, and analysis. The risk of bias is assessed in four domains, and the applicability is assessed in the first three domains. Each signalling question can be answered as “yes”, “probably yes”, “no”, “probably no” or “no information”. If at least one signalling question in a domain is answered as “no” or “probably no”, that domain should be considered at high risk of bias. The overall bias can only be considered low risk if all domains are judged to be low risk for bias.

| **General information** | | **Study** | | | | | | | | | | **High** | | **Low** | | **Unclear** | |
| --- | --- | --- | --- | --- | --- | --- | --- | --- | --- | --- | --- | --- | --- | --- | --- | --- | --- |
|  | | Piao 2021 | Wang 2018 | Zhang 2020 | Zhao 2020 | Yang 2022 | Wang 2022 | Liao 2021 | Wang 2023 | Hu 2021 | Chen 2024 | |  | |  | |  |
| **Domain 1: Participants** | 1.1 Were appropriate data sources used, e.g. cohort, RCT or nested case-control study data? | Yes | Yes | Yes | Yes | Yes | Yes | Yes | Yes | Yes | Yes | | 0 | | 10 | | 0 |
|  | Judgment Basis | Single-center retrospective cohort. | Single-center retrospective cohort. | Single-center retrospective cohort. | Single-center retrospective cohort. | Single-center retrospective cohort. | Single-center retrospective cohort. | Single-center retrospective cohort. | Single-center retrospective cohort. | Single-center retrospective cohort. | Single-center retrospective cohort. | |  | |  | |  |
|  | 1.2 Were all inclusions and exclusions of participants appropriate? | Yes | Yes | Yes | **Unclear** | Yes | Yes | Yes | Yes | Yes | Yes | | 0 | | 9 | | 1 |
|  | Judgment Basis | Inclusion/exclusion criteria clearly defined. | Inclusion/exclusion criteria clearly defined. | Inclusion/exclusion criteria clearly defined. | Exclusion criteria not explicitly stated | Inclusion/exclusion criteria clearly defined. | Inclusion/exclusion criteria clearly defined. | Inclusion/exclusion criteria clearly defined. | Inclusion/exclusion criteria clearly defined | Inclusion/exclusion criteria clearly defined. | Inclusion/exclusion criteria clearly defined. | |  | |  | |  |
|  | Domain-level RoB judgement | Low | Low | Low | **Unclear** | Low | Low | Low | Low | Low | Low | | 0 | | **9** | | **1** |
|  | Domain-level applicability judgement | Low | Low | Low | Low | Low | Low | Low | Low | Low | Low | | 0 | | **10** | | 0 |
| **Domain 2: Predictors** | 2.1 Were predictors defined and assessed in a similar way for all participants? | Yes | Yes | Yes | Yes | Yes | Yes | Yes | Yes | Yes | Yes | | 0 | | 10 | | 0 |
|  | Judgment Basis | Identical MRI protocols , feature extraction. | Identical MRI protocols , feature extraction. | Identical MRI protocols , feature extraction. | Identical MRI protocols , feature extraction. | Identical CT protocols , feature extraction. | Identical MRI protocols , feature extraction. | Identical MRI protocols , feature extraction. | Identical MRI protocols , feature extraction. | Identical MRI protocols , feature extraction. | Identical MRI protocols , feature extraction. | |  | |  | |  |
|  | 2.2 Were predictor assessment made without knowledge of outcome data? | Yes | Yes | Yes | Yes | Yes | Yes | Yes | Yes | Yes | Yes | | 0 | | 10 | | 0 |
|  | Judgment Basis | Tumor response assessments were performed without knowledge of the outcome data. | Tumor response assessments were performed without knowledge of the outcome data. | Tumor response assessments were performed without knowledge of the outcome data. | Tumor response assessments were performed without knowledge of the outcome data. | Tumor response assessments were performed without knowledge of the outcome data. | Tumor response assessments were performed without knowledge of the outcome data. | Tumor response assessments were performed without knowledge of the outcome data. | Tumor response assessments were performed without knowledge of the outcome data. | Tumor response assessments were performed without knowledge of the outcome data. | Tumor response assessments were performed without knowledge of the outcome data. | |  | |  | |  |
|  | 2.3 Are all predictors available at the time the model is intended to be used? | Yes | Yes | Yes | Yes | Yes | Yes | Yes | Yes | Yes | Yes | | 0 | | 10 | | 0 |
|  | Judgment Basis | Predictors were generated prior to treatment. | Predictors were generated prior to treatment. | Predictors were generated prior to treatment. | Predictors were generated prior to treatment. | Predictors were generated prior to treatment. | Predictors were generated prior to treatment. | Predictors were generated prior to treatment. | Predictors were generated prior to treatment. | Predictors were generated prior to treatment. | Predictors were generated prior to treatment. | |  | |  | |  |
|  | Domain-level RoB judgement | Low | Low | Low | Low | Low | Low | Low | Low | Low | Low | | 0 | | **10** | | 0 |
|  | Domain-level applicability judgement | Low | Low | Low | Low | Low | Low | Low | Low | Low | Low | | 0 | | **10** | | 0 |
| **Domain 3: Outcome** | 3.1 was the outcome determined appropriately? | Yes | Yes | Yes | Yes | Yes | Yes | Yes | Yes | Yes | Yes | | 0 | | 10 | | 0 |
|  | Judgment Basis **& E**vidence | RECIST 1.1 standard. | RECIST 1.1 standard. | RECIST 1.1 standard. | RECIST 1.1 standard. | RECIST 1.1 standard. | RECIST 1.1 standard. | RECIST 1.1 standard. | RECIST 1.1 standard. | RECIST 1.1 standard. | RECIST 1.1 standard. | |  | |  | |  |
|  | 3.2 Was a pre-specified or standard outcome definition used? | Yes | Yes | Yes | Yes | Yes | Yes | Yes | Yes | Yes | Yes | | 0 | | 10 | | 0 |
|  | Judgment Basis **& E**vidence | RECIST 1.1 standard. | RECIST 1.1 standard. | RECIST 1.1 standard. | RECIST 1.1 standard. | RECIST 1.1 standard. | RECIST 1.1 standard. | RECIST 1.1 standard. | RECIST 1.1 standard. | RECIST 1.1 standard. | RECIST 1.1 standard. | |  | |  | |  |
|  | 3.3 Were predictors excluded from the outcome definition? | Yes | Yes | Yes | Yes | Yes | Yes | Yes | Yes | Yes | Yes | | 0 | | 10 | | 0 |
|  | Judgment Basis | Outcome assessment independent of radiomics features. | Outcome assessment independent of radiomics features. | Outcome assessment independent of radiomics features. | Outcome assessment independent of radiomics features. | Outcome assessment independent of radiomics features. | Outcome assessment independent of radiomics features. | Outcome assessment independent of radiomics features. | Outcome assessment independent of radiomics features. | Outcome assessment independent of radiomics features. | Outcome assessment independent of radiomics features. | |  | |  | |  |
|  | 3.4 Was the outcome defined and determined in a similar way for all participants? | Yes | Yes | Yes | Yes | Yes | Yes | Yes | Yes | Yes | Yes | | 0 | | 10 | | 0 |
|  | Judgment Basis | Uniform MRI protocols and RECIST 1.1 applied to all patients. | Uniform MRI protocols and RECIST 1.1 applied to all patients. | Uniform MRI protocols and RECIST 1.1 applied to all patients. | Uniform MRI protocols and RECIST 1.1 applied to all patients. | Uniform CT protocols and RECIST 1.1 applied to all patients. | Uniform MRI protocols and RECIST 1.1 applied to all patients. | Uniform MRI protocols and RECIST 1.1 applied to all patients. | Uniform MRI protocols and RECIST 1.1 applied to all patients. | Uniform MRI protocols and RECIST 1.1 applied to all patients. | Uniform MRI protocols and RECIST 1.1 applied to all patients. | |  | |  | |  |
|  | 3.5 Was the outcome determine without knowledge of predictor information? | Yes | Yes | Yes | Yes | Yes | Yes | Yes | Yes | Yes | Yes | | 0 | | 10 | | 0 |
|  | Judgment Basis | RECIST 1.1 pre-specified. | RECIST 1.1 pre-specified. | RECIST 1.1 pre-specified. | RECIST 1.1 pre-specified. | RECIST 1.1 pre-specified. | RECIST 1.1 pre-specified. | RECIST 1.1 pre-specified. | RECIST 1.1 pre-specified. | RECIST 1.1 pre-specified. | RECIST 1.1 pre-specified. | |  | |  | |  |
|  | 3.6 Was the time interval between predictor assessment and outcome? | Yes | Yes | Yes | Yes | Yes | Yes | Yes | Yes | Yes | Yes | | 0 | | 10 | | 0 |
|  | Judgment Basis | The efficacy was evaluated 3 weeks after treatment. | The efficacy was evaluated 1 week after treatment. | The efficacy was evaluated 1 week after treatment. | The efficacy was evaluated 3 weeks after treatment. | Outcome assessed after 3 IC cycles (consistent timing). | Outcome assessed after 2 NAC cycles (42 days). | 1 week after the second cycle of ICT. | Outcome assessed 1 week post-chemotherapy. | Outcome assessed after two NACT cycles (21 days/cycle). | Outcome assessed 1 week post-ICT. | |  | |  | |  |
|  | Domain-level RoB judgement | Low | Low | Low | Low | Low | Low | Low | Low | Low | Low | | 0 | | **10** | | 0 |
|  | Domain-level applicability judgement | Low | Low | Low | Low | Low | Low | Low | Low | Low | Low | | 0 | | **10** | | 0 |
| **Domain 4: Analysis** | 4.1 Were there a reasonable number of participants with the outcome? | Yes | **No** | **No** | **No** | Yes | Yes | **No** | Yes | **No** | **No** | | 6 | | 4 | | 0 |
|  | Judgment Basis | EPV=26 | EPV=4.67<10 | EPV=6.25<10 | EPV=1.79<10 | EPV=19.88 | EPV=19.33 | EPV=9.17<10 | EPV=17 | EPV=9.56<10 | EPV=7<10 | |  | |  | |  |
|  | 4.2 Were continuous and categorical predictors handled appropriately? | Yes | Yes | Yes | Yes | Yes | Yes | Yes | Yes | Yes | Yes | | 0 | | 10 | | 0 |
|  | Judgment Basis | LASSO | LASSO | Z-score normalization for features; logistic regression for binary outcome. | LASSO + SVM | Continuous features standardized. | Continuous features processed via logistic regression. | Continuous features standardized. | mRMR + LASSO | LASSO | Continuous features standardized. | |  | |  | |  |
|  | 4.3 Were all enrolled participants included in the analysis? | Yes | Yes | Yes | Yes | Yes | Yes | Yes | Yes | Yes | Yes | | 0 | | 10 | | 0 |
|  | Judgment Basis | No reported exclusions post-enrollment. | No reported exclusions post-enrollment. | No reported exclusions post-enrollment. | No reported exclusions post-enrollment. | No reported exclusions post-enrollment. | No reported exclusions post-enrollment. | No reported exclusions post-enrollment. | No reported exclusions post-enrollment. | No reported exclusions post-enrollment. | No reported exclusions post-enrollment. | |  | |  | |  |
|  | 4.4. Were participants with missing data handled appropriately? | Yes | Yes | Yes | Yes | Yes | Yes | Yes | Yes | Yes | Yes | | 0 | | 10 | | 0 |
|  | Judgment Basis | No missing data reported. | No missing data reported. | No missing data reported. | No missing data reported. | No missing data reported. | No missing data reported. | No missing data reported. | No missing data reported. | No missing data reported. | No missing data reported. | |  | |  | |  |
|  | 4.5 Was selection of predictors based on univariable analysis avoided? | Yes | Yes | Yes | Yes | **No** | Yes | Yes | Yes | Yes | Yes | | 1 | | 9 | | 0 |
|  | Judgment Basis | LASSO | LASSO | Used mRMR + LASSO (multivariable approach). | LASSO | Features pre-selected via univariate analysis. | Stepwise forward selection using multivariate criteria (Spearman + MIC). | LASSO | mRMR + LASSO (multivariable approach) | mRMR + LASSO for multivariable feature selection. | Multivariate analysis was performed to select the significant clinical predictors. | |  | |  | |  |
|  | 4.6 Were complexities in the data (e.g., censoring, competing risks, sampling of control participants) accounted for appropriately? | Yes | Yes | Yes | Yes | Yes | Yes | Yes | Yes | Yes | Yes | | 0 | | 10 | | 0 |
|  | Judgment Basis | No complexities reported; standard methods applied. | No complexities reported; standard methods applied. | No complexities reported; standard methods applied. | No complexities reported; standard methods applied. | No complexities reported; standard methods applied. | No complexities reported; standard methods applied. | No complexities reported; standard methods applied. | No complexities reported; standard methods applied. | No complexities reported; standard methods applied. | No complexities reported; standard methods applied. | |  | |  | |  |
|  | 4.7 Were relevant model performance measures evaluated appropriately? | Yes | Yes | Yes | Yes | Yes | Yes | Yes | Yes | Yes | Yes | | 0 | | 10 | | 0 |
|  | Judgment Basis | AUC, sensitivity, specificity reported via ROC analysis. | AUC, sensitivity, specificity, PPV/NPV reported. | AUC, accuracy, sensitivity, specificity reported for training/validation cohorts. | Training: Cross-validation used; Validation: Small cohort inflated AUC confidence intervals. | AUC, sensitivity, specificity, F1-score reported. | AUC, sensitivity, specificity with bootstrapping and independent testing. | AUC, sensitivity, specificity reported. | Reported AUC, sensitivity, specificity. | AUC, DCA, Hosmer-Lemeshow test, and bootstrapping used | AUC, calibration, DCA reported. | |  | |  | |  |
|  | 4.8 Were model overfitting and optimism in model performance accounted for? | Probably Yes | Yes | Yes | Yes | Yes | Yes | Yes | Yes | Yes | Yes | | 0 | | 10 | | 0 |
|  | Judgment Basis | ANOVA + MW test, the correlation analysis, LASSO | Bootstrap validation | External validation | External validation | External validation | External validation | External validation | 10-fold CV, External validation | 10-fold CV, External validation | 10-fold CV, External validation | |  | |  | |  |
|  | 4.9 Do predictors and their assigned weights in the final model correspond to the results from the reported multivariable analysis? | Yes | Yes | Yes | Yes | Yes | Yes | Yes | Yes | Yes | Yes | | 0 | | 10 | | 0 |
|  | Judgment Basis | Two features selected via LASSO and used in logistic regression. | LASSO coefficients provided. | The feature coefficients (multivariable analysis results) perfectly align with the weights assigned in the final radiomics model. | Features (Rad-score coefficients) were based on the same regression model and aligned with the output of the feature selection method. | Features fed directly into model without post-hoc adjustments. | Logistic regression weights directly from multivariate analysis. | LASSO coefficients consistent with multivariable regression. | LASSO-selected features ( Rad-score, T stage) were fed directly into logistic regression without post-hoc adjustments. | The LASSO-selected features (Rad-score, T stage, LDH) were fed directly into logistic regression without post-hoc adjustments. | Rad-Score coefficients align with LASSO. | |  | |  | |  |
|  | Domain-level RoB judgement | Low | **High** | **High** | **High** | **High** | Low | **High** | Low | **High** | **High** | | **7** | | **3** | | 0 |
| **Overall assessment** | Overall judgement about RoB | Low | **High** | **High** | **High** | **High** | Low | **High** | Low | **High** | **High** | | **7** | | **3** | | 0 |
|  | Overall judgement about applicability | Low | Low | Low | Low | Low | Low | Low | Low | Low | Low | | 0 | | **10** | | 0 |

**Table S4** RQS of individual studies.

| **Study Criteria** | **Piao 2021** | **Wang 2022** | **Wang 2018** | **Zhang 2020** | **Zhao 2020** | **Yang 2022** | **Liao 2021** | **Wang 2023** | **Hu 2021** | **Chen 2024** |
| --- | --- | --- | --- | --- | --- | --- | --- | --- | --- | --- |
| 1. Image protocol quality | +2 | +2 | +2 | +2 | +0 | +2 | +2 | +2 | +2 | +2 |
| Judgment Basis | MRI scanning parameters (TR/TE/matrix size/slice thickness) are documented. | MRI protocols (T1, T2, T1-cs sequences) are explicitly documented (TR/TE values, matrix size, pixel spacing, slice spacing). | MR acquisition parameters (TR, TE, flip angle, slice thickness) are comprehensively documented. | MRI protocols documented (3T Siemens Verio, T1C sequence parameters) . | Scanner brands entioned, no parameter details. | Complete CT parameter documentation (120 kVp, 250 mAs, FOV 500×500 mm, 2 mm slice thickness) | Scan parameters (slice thickness, sequences) documented. | MRI protocols documented (slice thickness, sequence parameters) | MRI protocols (FOV, TR/TE, slice thickness) documented for T2WI and enhanced T1WI sequences. | MRI scanning protocols (including sequence parameters like TR, TE, matrix size) are well-documented in Methods section. |
| 1. Multiple segmentations | +1 | +1 | 0 | +1 | +1 | +1 | +1 | +1 | +1 | +1 |
| Judgment Basis | ROI segmentation was manually performed by two radiologists. | Two radiation oncologists performed segmentations; DSC ≥0.7 threshold enforced for inter-observer consistency. Feature robustness to segmentation variability analyzed. | Single radiologist performed ROI segmentation. No analysis of inter-observer variability or segmentation robustness. | Two radiologists performed manual segmentation; ICC >0.75 reported for inter-observer variability. | ROIs were delineated by three radiologists​with at least 10 years of clinical diagnosing experience. | Dual-radiologist manual segmentation. ICC > 0.85 for feature robustness. | Two radiologists independently segmented tumors; ICC >0.85. | Two radiologists performed segmentation with ICC validation (>0.75) | ROIs manually segmented by two radiologists (10/20 years’ experience); ICC > 0.75 reported. | ROIs manually segmented by two radiologists, with intraobserver/interobserver ICC evaluation to ensure feature robustness against segmentation variability. |
| 1. Phantom study on all scanners | 0 | 0 | 0 | 0 | 0 | 0 | 0 | 0 | 0 | 0 |
| Judgment Basis | No relevant description. | No relevant description. | No relevant description. | No relevant description. | No relevant description. | No relevant description. | No relevant description. | No relevant description. | No relevant description. | No relevant description. |
| 1. Imaging at multiple time points | 0 | 0 | 0 | 0 | 0 | 0 | 0 | 0 | 0 | 0 |
|  | No relevant description. | No relevant description. | No relevant description. | No relevant description. | No relevant description. | No relevant description. | No relevant description. | No relevant description. | No relevant description. | No relevant description. |
| 1. Feature reduction or adjustment for multiple testing | +3 | +3 | +3 | +3 | +3 | +3 | +3 | +3 | +3 | +3 |
| Judgment Basis | Triple feature selection: ANOVA/MW test → correlation analysis (threshold=0.9) → LASSO regression. | Stepwise forward selection reduced 31,920 features → 25 key features. Bootstrapping (1,000 samples) addressed overfitting. Feature robustness considered. | LASSO regression used for feature selection (from 591 features), reducing overfitting risk. | mRMR + LASSO for feature reduction; bootstrap validation (10,000 iterations). | Figure 1c and Methods: "Feature selection using two-sample t-test and LASSO regression. | Recursive Feature Elimination (RFE) framework. SVM-based stability selection. | LASSO feature selection + Bonferroni correction (p<0.01). | mRMR + LASSO feature selection with 10-fold cross-validation. | mRMR + LASSO with 10-fold cross-validation used for feature selection. | LASSO regression for feature selection + SMOTE for sample balancing implemented. Effectively reduces overfitting risk. |
| 1. Multivariable analysis with non-radiomics feature | 0 | 0 | 0 | 0 | +1 | +1 | +1 | +1 | +1 | +1 |
| Judgment Basis | No inclusion of non-radiomics features. | No inclusion of non-radiomics features. | No inclusion of non-radiomics features | No inclusion of non-radiomics features. | Radiomics nomogram integrating clinical factors. | Integrated clinical variables with radiomics. Clinical-radiological model AUC=0.933. | Integrated clinical variables with radiomics. | Combined radiomics with clinical T-stage in nomogram. | Combined radiomics (Rad-score) with clinical factors (T stage, LDH, Age). | Combined radiomics features with clinical-radiological factors (ICT regimens, T stage, enhancement degree) in logistic regression models. |
| 1. Detect and discuss biological correlates | 0 | 0 | 0 | +1 | +1 | 0 | 0 | 0 | +1 | +1 |
| Judgment Basis | No exploration of biological mechanisms underlying radiomics features. | No exploration of biological mechanisms underlying radiomics features. | No exploration of biological mechanisms underlying radiomics features. | Radiomic features reflect changes in the tumor microenvironment (e.g., cell density, necrotic regions). | Sex and the level of leukocytes before treatment were identified as independent predictors with significance for IC response. | No exploration of biological mechanisms underlying radiomics features. | No exploration of biological mechanisms underlying radiomics features. | No exploration of biological mechanisms underlying radiomics features. | Some reports have suggested that the serum LDH level is an independent prognostic factor in the treatment of non-distant metastasis. | Multivariate regression analyses demonstrated that ICT regimens , T stage , and enhanced degree were identified as the independent pre dictors for ICT response. |
| 1. Cutoff analyses | +1 | +1 | +1 | +1 | +1 | 0 | +1 | +1 | 0 | +1 |
| Judgment Basis | Cutoff values, sensitivity, and specificity reported for individual/combined features. | Continuous risk score g(x) converted to probability π(x); 0.5 used as response/non-response threshold. | Cut-off determined using ROC curve (maximal positive likelihood ratio) for Rad-score. | Optimal cut-off determined via Youden’s index; risk stratification applied. | Optimal cutoff value derived from Cox regression analysis. | No relevant description. | Optimal threshold determined by Youden index. | Optimal threshold determined by Youden index. | No relevant description. | Optimal cut-off determined via Youden’s index; risk stratification applied. |
| 1. Discrimination statistics | +1 | +2 | +2 | +2 | +2 | +2 | +2 | +2 | +2 | +2 |
|  | AUC, CI, and p-values reported , but no resampling (e.g., cross-validation). | AUC, sensitivity, specificity reported with 95% CIs and bootstrapping (1,000 samples) for cross-validation and independent testing. | AUC, sensitivity, specificity, PPV/NPV reported with 95% CIs and p-values. Bootstrap validation (1000 samples) performed. | AUC reported + 95% CI; bootstrap resampling. | Repeated 5-fold cross-validation with AUC and p-values reported. | Reported AUC, sensitivity, specificity (95% CI). Train-test validation | Reported AUC with 95% CI. 10-fold cross-validation. | AUC, sensitivity, specificity reported with 95% CIs. 10-fold cross-validation reported. | AUC, ROC, DeLong test, and 10-fold cross-validation reported. | AUC with 95% CI, sensitivity, specificity reported. Statistical significance validated through 10-fold cross-validation. |
| 1. Calibration statistic | 0 | 0 | 0 | 0 | +1 | +1 | +1 | +1 | +1 | +1 |
| Judgment Basis | No relevant description. | No relevant description. | No relevant description. | No relevant description. | Calibration curves | Calibration curves | Calibration curves | Calibration curves | Hosmer-Lemeshow test (p > 0.05) for nomogram calibration. | Calibration curves |
| 1. Prospective study registered in a trial database | 0 | 0 | 0 | 0 | 0 | 0 | 0 | 0 | 0 | 0 |
| Judgment Basis | No relevant description. | No relevant description. | No relevant description. | No relevant description. | No relevant description. | No relevant description. | No relevant description. | No relevant description. | No relevant description. | No relevant description. |
| 1. Validation | -5 | +2 | +2 | +2 | +2 | +2 | +2 | +2 | +2 | +2 |
| Judgment Basis | No external validation. | Internal validation (cross-validation cohort, n=85) and independent testing (testing cohort, n=80) performed. | Internal validation using bootstrap resampling (1000 samples) on the same dataset. | Internal validation only (single-institute 70:30 split). | Validation cohort from the same institution. | Internal validation cohort from same institution. | Internal validation cohort from same institution. | Internal validation cohort from same institution. | Internal validation cohort from same institution. | Internal validation using 10-fold cross-validation on single-institution data. |
| 1. Comparison to “gold standard” | +2 | +2 | +2 | +2 | +2 | +2 | +2 | +2 | +2 | +2 |
| Judgment Basis | Tumor response was evaluated before treatment and after completing. According to the RECIST 1.1 criteria. | Tumor response was evaluated before treatment and after completing. According to the RECIST 1.1 criteria. | Tumor response was evaluated before treatment and after completing. According to the RECIST 1.1 criteria. | Tumor response was evaluated before treatment and after completing. According to the RECIST 1.1 criteria. | Tumor response was evaluated before treatment and after completing. According to the RECIST 1.1 criteria. | Tumor response was evaluated before treatment and after completing. According to the RECIST 1.1 criteria. | Tumor response was evaluated before treatment and after completing. According to the RECIST 1.1 criteria. | Tumor response was evaluated before treatment and after completing. According to the RECIST 1.1 criteria. | Tumor response was evaluated before treatment and after completing. According to the RECIST 1.1 criteria. | Tumor response was evaluated before ICT treatment and 1 week after completing two to three cycles of ICT. According to the RECIST 1.1 criteria |
| 1. Potential clinical utility | 0 | 0 | 0 | 0 | +2 | +2 | +2 | +0 | +2 | +2 |
| Judgment Basis | No relevant description. | No relevant description. | No relevant description. | DCA for net benefit assessment.strategy. | DCA for net benefit assessment. | DCA for net benefit assessment. | DCA for net benefit assessment. | DCA for net benefit assessment. | DCA for net benefit assessment. | DCA for net benefit assessment. |
| 1. Cost-effectiveness analysis | 0 | 0 | 0 | 0 | 0 | 0 | 0 | 0 | 0 | 0 |
| Judgment Basis | No relevant description. | No relevant description. | No relevant description. | No relevant description. | No relevant description. | No relevant description. | No relevant description. | No relevant description. | No relevant description. | No relevant description. |
| 1. Open science and data | +2 | +2 | +2 | +2 | +1 | +2 | +2 | +2 | +2 | +2 |
| Judgment Basis | MRI acquisition protocol. Image preprocessing and tumor segmentation. | MRI acquisition protocol. Image preprocessing and tumor segmentation. | MRI acquisition protocol. Image preprocessing and tumor segmentation. | Radiomics features published in supplementary data. | MRI acquisition protocol. (No specific details of the agreement) | CT acquisition protocol. Image preprocessing and tumor segmentation. Code publicly available (GitHub). | MRI acquisition protocol. Image preprocessing and tumor segmentation. | MRI acquisition protocol. Image preprocessing and tumor segmentation. | MRI acquisition protocol. Image preprocessing and tumor segmentation. | MRI acquisition protocol. Image preprocessing and tumor segmentation. |
| Total score (Maximum:36) | 7 | 15 | 14 | 16 | 17 | 18 | 19 | 17 | 19 | 20 |

Table S5 Adherence rate of IBSI pre-processing steps

| **Pre-processing performed** | **Piao 2021** | **Wang 2022** | **Wang 2018** | **Zhang 2020** | **Zhao 2020** | **Yang 2022** | **Liao 2021** | **Wang 2023** | **Hu 2021** | **Chen 2024** | **Number of studies**  **(adherence rate, %)** |
| --- | --- | --- | --- | --- | --- | --- | --- | --- | --- | --- | --- |
| **Intensity normalization** | Yes | **No** | Yes | Yes | Yes（Auxiliary materials provision） | **No** | Yes | **No** | **No** | **No** | 5 (50) |
| Judgment Basis | Z-score normalization was explicitly applied to standardize texture features. | No relevant description. | Explicit mathematical formula provided with parameters. | z-score normalization was used. | Intensity normalization deformed intensities of images. | No relevant description. | Z-score standardization explicitly applied to all radiomics features. | No relevant description. | No relevant description. | No relevant description. |  |
| **Segmentation method** | Yes | Yes | Yes | Yes | Yes | Yes | Yes | Yes | Yes | Yes | 10 (100) |
| Judgment Basis | Manual segmentation using ITK-SNAP software was performed by two deputy chief radiologists, including details on expertise and ROI delineation. | Manual segmentation by two experts; DSC validation and consensus strategy. | Manual ROI delineation by a single radiologist (10+ years experience) using ImageJ; cross-sequence replication described. | Manual segmentation with two experts and ICC validation. | Manual segmentation of tumors and region of interest (ROIs) were delineated by three  radiologists with at least 10 years of clinical diagnosing experience T2-WI using MITK . | Manual segmentation by two radiologists. Interobserver ICC validation (ICC > 0.85). | Manual segmentation by two radiologists (8/12 years experience), with senior arbitration (25 years). Inter-observer ICC reported (0.85–0.94). | Manual segmentation of VOIs was delineated by a junior radiologist, with 5 years of head and neck cancer diagnosing experience, using the ITKSNAP software. | ROI delineation manually performed by two radiologists (10-/20-year experience) with inter-/intra-observer ICC validation (ICC > 0.75). | Manual slice-by-slice segmentation using 3D-Slicer by two radiologists (5/10 years of experience), with senior review for consensus. ICC evaluated for intra-/inter-observer reliability. |  |
| **Image interpolation** | **No** | **No** | **No** | **No** | **No** | Yes | Yes | Yes | **No** | Yes | 4 (40) |
| Judgment Basis | No relevant description. | No relevant description. | No relevant description. | No relevant description. | No relevant description. | Resampling to 1×1×1 mm³ voxels mentioned. | Resampling to 1×1×1 mm³ voxels confirmed. | Voxel resampling is the resampling of all images to a voxel size of 1x1x1mm³. | No relevant description. | Linear interpolation used to resample images to 1×1×1 mm³ isotropic voxels, standardizing voxel spacing. |  |
| **Grey-level discretization** | **No** | Yes | **No** | **No** | **No** | Yes | Yes | Yes | **No** | **No** | 4 (40) |
| Judgment Basis | No relevant description. | Specifies gray-level quantization (GL: 8-64) and algorithms (Equal/Lloyd). | No relevant description. | No relevant description. | No relevant description. | Bin width of image intensities as 25 HU. | Uniform binwidth discretization (bin size=25) explicitly stated. | To discretise the image intensities, the bin number(32 bins) of all the pre-treatment MRI images was fixed. | No relevant description. | No relevant description. |  |
| **Image filter** | **No** | Yes | Yes | **No** | Yes | Yes | Yes | Yes | Yes | Yes | 8(80) |
| Judgment Basis | No relevant description. | Uses wavelet band-pass filters with defined weights (WW: 0.5–2.0). | LoG filter fully defined. | No relevant description. | Features in the first three groups were calculated for each of 8 wavelet decompositions. | Wavelet features extracted via high/low-pass filters. | Wavelet filtration extracted. | Gaussian denoising. | Wavelet features extracted (implicit filtering). | No relevant description. |  |
| **IBSI compliance** | **No** | **No** | **No** | **No** | **No** | Yes | Yes | Yes | **No** | Yes | 4 (40) |
| Judgment Basis | No relevant description. | No relevant description. | No relevant description. | No relevant description. | No relevant description. | PyRadiomics package used for feature extraction, which complies with IBSI guidelines. | PyRadiomics package used for feature extraction, which complies with IBSI guidelines. | The majority of features abide by feature definitions laid out by IBSI. | No relevant description. | PyRadiomics package used for feature extraction, which complies with IBSI guidelines. |  |
| **Robustness assessment** | **No** | Yes | **No** | Yes | **No** | Yes | Yes | Yes | Yes | Yes | 7 (70) |
| Judgment Basis | No relevant description. | Bootstrap validation (1,000 samples) and imbalance adjustment for small samples. | No relevant description. | ICC and Bootstrap methods used. | No relevant description. | ICC > 0.85 for interobserver features. | Inter-observer ICC >0.85, assessed for segmentation stability. | The same delineation method and software were used to calculate the intraclass correlation coefficient (ICC). | Segmentation consistency assessed via ICC. | Intraclass Correlation Coefficient (ICC) evaluation for manual segmentation.10-fold cross-validation integrated into LASSO regression. |  |
| **Total** |  | | | | | | | | | | 42 (70) |

**Table S6** League table of the three outcome indicators.

| **League table on Sensitivity *I*^2^ =78.31%** | | | | | | | | |
| --- | --- | --- | --- | --- | --- | --- | --- | --- |
| MRI SVM-Clinical | 0.61 (0.00,93.64) | 0.18 (0.01,3.98) | 0.08 (0.00,11.18) | 0.10 (0.00,6.43) | 0.10 (0.00,6.64) | 0.02 (0.00,3.10) | 0.02 (0.00,2.08) | 0.01 (0.00,1.72) |
| 1.65 (0.01,255.78) | MRI FCNN | 0.29 (0.00,23.69) | 0.13 (0.00,9.43) | 0.17 (0.01,3.39) | 0.17 (0.01,3.33) | 0.04 (0.00,2.59) | 0.03 (0.00,1.74) | 0.02 (0.00,1.44) |
| 5.65 (0.25,126.87) | 3.42 (0.04,276.41) | MRI SVM | 0.45 (0.01,32.49) | 0.59 (0.02,15.91) | 0.58 (0.02,16.65) | 0.13 (0.00,8.94) | 0.09 (0.00,6.00) | 0.07 (0.00,4.96) |
| 12.47 (0.09,1738.14) | 7.54 (0.11,536.80) | 2.21 (0.03,158.47) | CT CNN | 1.30 (0.06,29.54) | 1.27 (0.05,31.03) | 0.29 (0.10,0.86) | 0.19 (0.07,0.57) | 0.16 (0.05,0.47) |
| 9.55 (0.16,587.33) | 5.78 (0.29,113.34) | 1.69 (0.06,45.57) | 0.77 (0.03,17.35) | MRI Radiomics | 0.97 (0.39,2.45) | 0.22 (0.01,4.69) | 0.15 (0.01,3.14) | 0.12 (0.01,2.60) |
| 9.81 (0.15,638.83) | 5.94 (0.30,117.30) | 1.74 (0.06,50.28) | 0.79 (0.03,19.22) | 1.03 (0.41,2.58) | MRI Radiomics-Clinical | 0.23 (0.01,5.20) | 0.15 (0.01,3.48) | 0.13 (0.01,2.88) |
| 43.25 (0.32,5788.44) | 26.17 (0.39,1776.10) | 7.66 (0.11,524.37) | **3.47 (1.16,10.38)** | 4.53 (0.21,96.02) | 4.41 (0.19,101.04) | CT SVM | 0.67 (0.28,1.61) | 0.56 (0.23,1.33) |
| 64.18 (0.48,8560.11) | 38.84 (0.57,2625.02) | 11.37 (0.17,775.00) | **5.15 (1.75,15.15)** | 6.72 (0.32,141.68) | 6.54 (0.29,149.12) | 1.48 (0.62,3.56) | CT SVM-Clinical | 0.83 (0.35,1.94) |
| 77.44 (0.58,10322.00) | 46.86 (0.69,3164.99) | 13.72 (0.20,934.43) | **6.21 (2.12,18.23)** | 8.11 (0.38,170.78) | 7.89 (0.35,179.75) | 1.79 (0.75,4.28) | 1.21 (0.52,2.82) | CT SVM-CNN |
| **League table on Specificity *I*^2^ =74.55%** | | | | | | | | |
| MRI SVM | 0.30 (0.01,10.94) | 0.15 (0.01,1.63) | 0.09 (0.00,7.28) | 0.09 (0.00,7.28) | 0.11 (0.00,3.91) | 0.10 (0.00,3.29) | 0.06 (0.00,5.00) | 0.05 (0.00,3.86) |
| 3.36 (0.09,123.18) | MRI FCNN | 0.51 (0.02,16.51) | 0.29 (0.01,6.40) | 0.29 (0.01,6.40) | 0.37 (0.18,0.75) | 0.32 (0.16,0.64) | 0.20 (0.01,4.39) | 0.15 (0.01,3.39) |
| 6.60 (0.61,71.09) | 1.97 (0.06,63.84) | MRI SVM-Clinical | 0.57 (0.01,43.51) | 0.57 (0.01,43.51) | 0.73 (0.02,22.82) | 0.63 (0.02,19.18) | 0.39 (0.01,29.89) | 0.30 (0.00,23.06) |
| 11.60 (0.14,979.12) | 3.46 (0.16,76.46) | 1.76 (0.02,134.36) | CT SVM-Clinical | 1.00 (0.42,2.39) | 1.28 (0.06,27.22) | 1.10 (0.05,22.80) | 0.69 (0.30,1.61) | 0.53 (0.23,1.23) |
| 11.60 (0.14,979.12) | 3.46 (0.16,76.46) | 1.76 (0.02,134.36) | 1.00 (0.42,2.39) | CT SVM-CNN | 1.28 (0.06,27.22) | 1.10 (0.05,22.80) | 0.69 (0.30,1.61) | 0.53 (0.23,1.23) |
| 9.09 (0.26,323.07) | **2.71 (1.34,5.48)** | 1.38 (0.04,43.25) | 0.78 (0.04,16.71) | 0.78 (0.04,16.71) | MRI Radiomics-Clinical | 0.86 (0.50,1.48) | 0.54 (0.03,11.45) | 0.42 (0.02,8.83) |
| 10.53 (0.30,364.66) | **3.14 (1.55,6.33)** | 1.59 (0.05,48.77) | 0.91 (0.04,18.78) | 0.91 (0.04,18.78) | 1.16 (0.68,1.98) | MRI Radiomics | 0.63 (0.03,12.87) | 0.48 (0.02,9.92) |
| 16.80 (0.20,1411.95) | 5.01 (0.23,110.04) | 2.55 (0.03,193.73) | 1.45 (0.62,3.38) | 1.45 (0.62,3.38) | 1.85 (0.09,39.17) | 1.60 (0.08,32.80) | CT CNN | 0.77 (0.34,1.74) |
| 21.74 (0.26,1823.75) | 6.48 (0.30,142.03) | 3.29 (0.04,250.22) | 1.87 (0.81,4.34) | 1.87 (0.81,4.34) | 2.39 (0.11,50.56) | 2.07 (0.10,42.34) | 1.29 (0.57,2.92) | CT SVM |
| **League table on Accuracy *I*^2^ =69.51%** | | | | | | | | |
| MRI SVM | 0.50 (0.02,14.37) | 0.74 (0.14,3.89) | 0.13 (0.00,8.73) | 0.18 (0.01,4.94) | 0.17 (0.01,4.49) | 0.08 (0.00,5.44) | 0.07 (0.00,4.94) | 0.07 (0.00,4.71) |
| 2.00 (0.07,57.26) | MRI FCNN | 1.48 (0.05,40.90) | 0.25 (0.01,6.06) | 0.37 (0.15,0.89) | 0.35 (0.14,0.83) | 0.16 (0.01,3.77) | 0.14 (0.01,3.42) | 0.14 (0.01,3.27) |
| 1.35 (0.26,7.10) | 0.68 (0.02,18.75) | MRI SVM-Clinical | 0.17 (0.00,11.48) | 0.25 (0.01,6.44) | 0.23 (0.01,5.85) | 0.11 (0.00,7.15) | 0.10 (0.00,6.50) | 0.09 (0.00,6.20) |
| 7.88 (0.11,542.71) | 3.95 (0.17,94.49) | 5.83 (0.09,390.52) | CT CNN | 1.45 (0.06,32.37) | 1.36 (0.06,29.35) | 0.63 (0.25,1.55) | 0.57 (0.23,1.40) | 0.54 (0.22,1.34) |
| 5.44 (0.20,146.14) | **2.72 (1.13,6.60)** | 4.02 (0.16,104.32) | 0.69 (0.03,15.41) | MRI Radiomics-Clinical | 0.94 (0.52,1.71) | 0.43 (0.02,9.58) | 0.39 (0.02,8.70) | 0.37 (0.02,8.30) |
| 5.78 (0.22,150.22) | **2.90 (1.20,6.99)** | 4.28 (0.17,107.19) | 0.73 (0.03,15.80) | 1.06 (0.58,1.94) | MRI Radiomics | 0.46 (0.02,9.82) | 0.42 (0.02,8.92) | 0.40 (0.02,8.51) |
| 12.61 (0.18,864.32) | 6.32 (0.27,150.27) | 9.33 (0.14,621.93) | 1.60 (0.65,3.96) | 2.32 (0.10,51.47) | 2.18 (0.10,46.67) | CT SVM-Clinical | 0.91 (0.38,2.20) | 0.87 (0.36,2.10) |
| 13.87 (0.20,950.08) | 6.95 (0.29,165.15) | 10.26 (0.15,683.63) | 1.76 (0.71,4.34) | 2.55 (0.11,56.56) | 2.40 (0.11,51.30) | 1.10 (0.45,2.66) | CT SVM-CNN | 0.95 (0.40,2.30) |
| 14.53 (0.21,995.33) | 7.28 (0.31,173.00) | 10.75 (0.16,716.19) | 1.84 (0.75,4.55) | 2.67 (0.12,59.25) | 2.51 (0.12,53.73) | 1.15 (0.48,2.79) | 1.05 (0.43,2.53) | CT SVM |

Note: Statistical significance was given in bold and established when the 95% CI did not cover 1.

**Table S7** Investigation of heterogeneity using meta-regression.

| **Parameter** | **Joint Model Analysis** | | |
| --- | --- | --- | --- |
|  | ***P*** | ***I²*** | **LRT chi^2^** |
| EPV | 0.07 | 63.0 (16.0, 100.0) | 5.39 |
|  |  |  |  |
| Device type | **﹤0.01** | 82.0 (61.0, 100.0) | 10.85 |
|  |  |  |  |
| ROI | 0.37 | 0.0 (0.0, 100.0) | 1.99 |
|  |  |  |  |
| Validation | 0.37 | 0.0 (0.0, 100.0) | 1.99 |
|  |  |  |  |
| Algorithms | 0.40 | 0.0 (0.0, 100.0) | 1.81 |
|  |  |  |  |
| Feature | 0.07 | 63.0 (16.0, 100.0) | 5.39 |
|  |  |  |  |
| RQS | 0.23 | 31.0 (0.0, 100.0) | 2.90 |
|  |  |  |  |
| Sample | **0.02** | 75.0 (44.0, 100.0) | 7.88 |
|  |  |  |  |

Note: EPV, events per variable; ROI, region of interest; RQS, radiomics quality score.


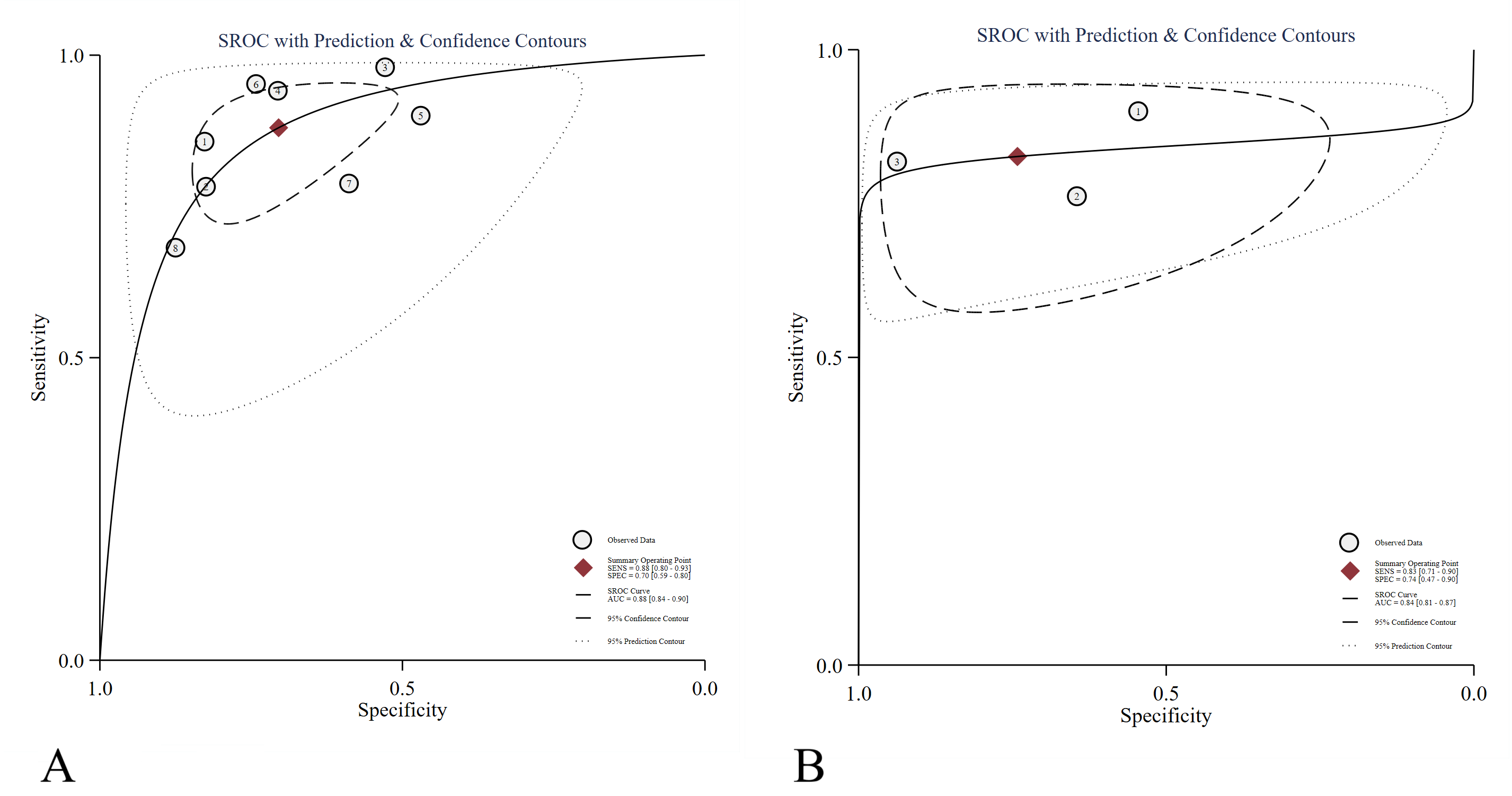


**Figuer S1** Single model predictive performance (SROC curve).

A, MRI Radiomics model; B, MRI Radiomics-Clinical model.
